# Integrated approach of extreme learning machines and locally weighted linear regression for improved discharge coefficient prediction

**DOI:** 10.1038/s41598-025-03812-z

**Published:** 2025-07-01

**Authors:** Mohammed Majeed Hameed, Mohamed Khalid Alomar, Siti Fatin Mohd Razali, Ali Salem

**Affiliations:** 1 Upper Euphrates Centre for Sustainable Development Research, University of Anbar, Ramadi City, 31001 Iraq; 2 Department of Civil Engineering, College of Engineering, University of Al-Maarif, Ramadi City, 31001 Iraq; 3 Department of Civil Engineering, Faculty of Engineering and Built Environment, Universiti Kebangsaan Malaysia, Bangi, 43600 Selangor Malaysia; 4 Smart and Sustainable Township Research Centre (SUTRA), Universiti Kebangsaan Malaysia, Bangi, 43600 Selangor Malaysia; 5 Civil Engineering Department, Faculty of Engineering, Minia University, Minia, 61111 Egypt; 6 Structural Diagnostics and Analysis Research Group, Faculty of Engineering and Information Technology, University of Pécs, Pécs, Hungary

**Keywords:** Discharge coefficient, Side weirs, Extreme learning machines, Locally weighted linear regression, Sensitivity analysis, Hydrology, Civil engineering, Hydrogeology

## Abstract

Accurate determination of the discharge coefficient (*Cd*) is essential for calculating discharge over side weirs. The current study aims to enhance the prediction accuracy of *Cd* for rectangular sharp-crested side weirs by addressing the limitation of the output layer of the Extreme learning machine (ELM). The output layer of ELM depends mainly on the linear system which limits its generalization capabilities. Therefore, this study uses Locally Weighted Linear Regression (LWLR) with radial basis kernel function instead of the linear system to effectively capture nonlinear relationships and enhance local data pattern recognition. The proposed model (ELM-LWLR) has been validated against classic multiple linear regression (MLR), ELM, LWLR, and Extreme Gradient Boosting (XGBoost). The quantitative results showed that the ELM-LWLR model has a superior performance, achieving higher prediction accuracy with a correlation coefficient of 0.968, and percentage bias (*PBIAS*) of -0.130%. Moreover, the accuracy of Cd prediction using the ELM-LWLR model improved by 37.21% compared to LWLR, 28.95% compared to XGBoost, 48.08% compared to ELM, and 64.94% compared to MLR. Additionally, sensitivity analysis identified the ratio of weir height to length and dimensionless length as critical factors affecting *Cd* estimation. Overall, the findings demonstrate that the ELM-LWLR model is a practical and robust tool for Cd modeling, offering significant advantages in cost reduction and enhanced hydraulic modeling for complex engineering applications.

**Supplementary Information:**

The online version contains supplementary material available at 10.1038/s41598-025-03812-z.

## Introduction

### Background

Side weirs are hydraulic structures primarily designed to divert flow^1,2^, commonly used in environmental engineering, river engineering, and hydraulics, and they also serve as a key regulator. The flow within the side weirs is complex and characterized by a spatial variation with diminishing discharge. Side weir structures have also been practically used to manage and discharge excess stormwater from irrigation canals, urban drainage, wastewater treatment facilities, and other systems^3^. Furthermore, in light of recent shifts in extreme weather patterns and the significant increase in storm flooding, side weirs have become a common solution in both sewer networks and irrigation systems^4^. These structures are used to redirect excess water flow from one channel to another. To maximize crest length in narrow channels for improving water discharge performance, weirs can be designed in various shapes, including trapezoidal, triangular, parabolic, diagonal, and rectangular^5,6^. In water resources management, precise estimation of the discharge coefficient (*Cd*) of weirs and its influencing parameters is critical for designing and operating such significant hydraulic structures.

### Prediction *Cd* using empirical equations

As *Cd* is a key factor in accurately computing the discharge, several works from the past few decades ago developed empirical equations. The developed equations usually depend on the geometry of the weirs and the hydraulic parameters of the flow. Some researchers have identified dozens of equations developed in literature^7^, focusing on incorporating hydraulic, flow, and geometric elements such as Froude number, crest height, flow depth, main channel width, and crest length and side. Although these equations consider the key parameters that influence *Cd*, they lack high precision, and their calibration requires relatively large experimental sample sizes, making them costly^8^. Additionally, these mathematical models were developed based on specific samples tested in hydraulic laboratories, making them incompatible when applied to conditions outside the conditions in which they were calibrated. However, despite their limitations in providing more accurate predictions of *Cd* due to the complexity of several factors affecting *Cd*, such as flow variability and geometry, these equations are still easy to implement and typically do not require further advanced computing.

### Prediction cd using data-driven models

Recently, data-driven models have garnered the attention of researchers in water resources and hydraulic fields^9,10^. These models effectively capture complex interactions within data, making them highly useful for solving engineering problems. Additionally, they are flexible and adaptable, allowing for adjustments based on new data and trends without requiring a complete overhaul. Many AI-based models have been developed in the hydraulic field to address engineering challenges and estimate the Cd^11–15^. For instance, stacking regression models like Extreme Gradient Boosting)XGBoost) demonstrate robust performance in estimating the *Cd* in broad-crested gabion weirs^16^. Another study investigated the capacity of Artificial Neural Networks (ANN) to estimate the *Cd* for oblique sluice gates^17^. The ANN was validated against several models, including Support Vector Regression (SVR), Random Forest (RF), Gaussian Processes (GP), and generalized regression neural networks (GRNN). The results highlighted that the ANN provided more accurate estimates of *Cd* compared to the other models.

Extreme learning machine (ELM), a newer variant of artificial neural networks, has been extensively studied for estimating the *Cd* for rectangular sharp-crested side weirs. The results indicate that ELM provides highly reliable and accurate estimations^18,19^. It is significant to highlight that the ELM has several outstanding advantages over standard ANN. It can be trained more quickly, demonstrates better generalization capacity, and has a lower computational cost^20^. Other researchers investigate three data-driven models to predict the *Cd* of streamlined weirs using machine learning techniques, including RF, Adaptive Network-Based Fuzzy Inference System (ANFIS), and Gene Expression Programming (GEP)^21^. The GEP model demonstrates the best accuracy, closely matching measured values within 0–10%. In hydraulic areas, ELM has also been utilized to estimate *Cd* for triangular labyrinth weirs and validated against ANN and genetic programming (GP) models^22^.

The key findings reveal that the ELM model is the most effective, producing *Cd* estimates that closely align with experimental results. Other researchers also constructed a hybrid model by integrating Grey Wolf algorithm (GWA) with kernel ELM (KELM) to enhance the prediction of *Cd.* The KELM based GWA has been validated against SVR and Gaussian process regression (GPR) and showed a significant improvement in the prediction accuracy^23^. Also, several researchers in the water resources sector have found that hybrid models incorporating optimization algorithms offer better predictive accuracy and more stable performance compared to classical models^24–27^. It is significant to note that numerous researchers have utilized ELM to tackle data-related problems in various scientific disciplines, particularly in the water resources field^28–32^.

### Problem statement and research motivation

Despite the widespread use of the ELM model in water resource management, its development has been limited, focusing mainly on optimizing weights and biases in the hidden layer^33–36^. Moreover, in previous works the hidden layer parameters of the ELM model have been optimized using several heuristics algorithms such as (GWA)^23^, a combination of Particle Swarm Optimization (PSO) and GWA^36,37^, Beluga Whale Optimization and Genetic Algorithm (GA)^35^, and Imperialist Competitive Algorithm^38^. Although hybrid ELM models may enhance the prediction capability, they predominantly concentrate on tuning the hidden layer parameters of ELM and neglect improvements in the output layer, which is accountable for producing the final outcomes. The output layer of ELM depends mainly on a linear system that may encounter difficulties with intricate engineering problems. Furthermore, the applied hybrid models are classified as global models, implying they may neglect regional data patterns. Moreover, these sophisticated hybrid models typically require complicated tuning of parameters and increased computing costs, which may not always result in substantial enhancements in accuracy.

To address these limitations, the current work investigates an alternative approach for computing the weights of the layer of ELM by using Locally Weighted Linear Regression (LWLR) instead of the classic linear system. The linear regression (LR) approach may not be able to accurately predict values for out-of-sample data points (i.e., data not used in the training phase). However, the LWLR-based model considers the local characteristics of the data, allowing for more accurate predictions on these out-of-sample data points. This may be particularly useful in real-world applications where new data is constantly being collected, and the model needs to adapt and make accurate predictions. Furthermore, LR also operates on a linear basis, a characteristic that might render it incapable of adequately detecting non-linear trends within the data. On the other hand, the LWLR, with its non-linear nature, is well-suited to tackle intricate engineering obstacles. This quality has enabled it to surpass some of the sophisticated AI models in past research^40^.

The current study incorporated ELM with LWLR for enhancing the predicting *Cd* of rectangular sharp-crested side weirs using an experimental dataset of more than 160 samples. The use of a classical linear regression system in the output layer in the ELM model^39^ can be a significant drawback, as it limits the model’s ability to capture complex, non-linear relationships between the inputs and outputs, thereby affecting its generalization capacity. Also, the linear system may contribute to the poor stability of the model in dealing with complex water problems and thus reduce the model’s ability to generalize. Thus, the adopted ELM-LWLR takes advantage of both the global model (ELM) and the nonlinear local model (LWLR) to capture the nonlinear relations in the prediction of *Cd.* In comparison to the other discharge coefficient prediction approaches (hybrid ELM models), the ELM-LWLR model has significant advantages. The LWLR approach used allows the model to incorporate localized data fluctuations, improving accuracy in complex hydraulic interactions, unlike classic ELM and hybrid ELM models, which use global linear regression in the output layer. Also, the ELM-LWLR model serves as an effective *Cd* model for varying hydraulic conditions, requiring less parameter tuning compared to bio-inspired hybrid models. Thus, the objectives of this paper can be summarized as below:

i. To enhance the output layer efficiency of ELM by using LWLR, thereby establishing a hybrid ELM-LWLR model for accurately predicting the *Cd* of rectangular sharp-crested side weirs using over 160 samples collected under varying flow conditions.
ii. To validate the performance of the proposed ELM-LWLR model against several prediction models (classic ELM, XGBoost, MLR, and LWLR), and other empirical and AI models developed in previous works.
iii. To conduct a detailed sensitivity analysis to identify the most effective hydraulic and geometric parameters influencing *Cd.*

## Data collection

The present study utilized experimental data from two notable research published in the literature^41,42^. The first study was conducted at the hydraulics laboratory of Firat University, utilizing a 12-meter-long rectangular channel for testing. A total of 79 samples were collected from this investigation. Notably, key geometric parameters like channel width, depth, and gradient were set at 0.5 m, 0.5 m, and 0.01, respectively. Precise measurements were taken using a high-precision Mitutoyo digital point micrometer, and water flow was meticulously controlled through a sluice gate. To ensure precise discharge readings, the team used both a V-notched weir and a standard rectangular weir for calibration.

The second research conducted experiments on rectangular sharp-crested weirs of varying sizes within an 8-meter-long rectangular channel, using dimensions of 0.4 m wide and 0.6 m deep^42^. A total of 83 samples were collected, with the team maintaining subcritical flow conditions throughout the study. For accurate measurements, a point meter mounted on a mobile carriage was employed. The researchers calculated the flow diverted from the weirs by comparing upstream and downstream discharges, a reliable method that yielded consistent results. Both research teams utilized an electromagnetic flow meter to measure discharge, with data ranges detailed in their respective publications. In both experimental setups, the sluice gate played a crucial role as a central control element for managing flow depth and downstream discharge, having been calibrated in advance to ensure maximum precision.

**Table 1:** Statistical analysis of the parameters used and the *Cd* coefficient.

Dataset	Variable	*X*_min_	*X*_mean_	*X*_med_	*X*_max_	*X*_skew_	*X*_std_	*X*_kur_	R*
Training	*p*/*y*1	0.181	0.637	0.644	0.910	−0.290	0.182	2.237	−0.440
	*F*1	0.088	0.359	0.318	0.771	0.401	0.160	2.182	−0.155
	*b*/*y*1	0.353	2.248	2.226	6.554	0.571	1.431	2.576	0.153
	*b*/*B*	0.300	0.931	1.008	1.513	−0.091	0.501	1.343	0.300
	*Cd*	0.285	0.483	0.490	0.846	0.487	0.093	4.063	1.000
Testing	*p*/*y*1	0.242	0.600	0.600	0.900	0.028	0.169	2.076	−0.573
	*F*1	0.119	0.384	0.364	0.804	0.596	0.165	2.799	−0.186
	*b*/*y*1	0.347	2.103	1.866	5.478	0.560	1.408	2.230	0.110
	*b*/*B*	0.300	0.884	0.760	1.513	0.105	0.488	1.412	0.215
	*Cd*	0.281	0.488	0.498	0.738	0.241	0.104	2.948	1.000
Overall	*p*/*y*1	0.181	0.635	0.641	0.91	−0.31	0.1804	−0.73	−0.120
	*F*1	0.088	0.365	0.34	0.804	0.47	0.1623	−0.56	−0.166
	*b*/*y*1	0.347	2.188	2.083	6.554	0.57	1.417	−0.45	0.372
	*b*/*B*	0.3	0.924	1.008	1.513	−0.06	0.5033	−1.69	0.206
	*Cd*	0.281	0.485	0.4925	0.846	0.53	0.097	0.89	1.000

Note: The terms *X*_*min*,_ *X*_*mean*_ *X*_*med*_ *X*_*max*_ *X*_*skew*_ *X*_*std*_ *X*_*kur*_ are respectively the minimum, average, median, maximum, skewness, standard deviation, and kurtosis. The R* is a linear correlation coefficient between each variable with the *Cd.*

A total of 162 samples were collected, encompassing *Cd*, as well as geometric, water, and hydraulic parameters^7^ such as the ratio of weir height to length (*p*/*y*1), the ratio of weir length to the depth of upstream flow (*b*/*y*1), dimensionless weir length (*b*/*B*), and the Froude number (*F*1). Descriptive statistics for these parameters are outlined in Table 1. It is noted that the linear relationship between *Cd* and the independent variables is weak, with very low correlation coefficients (*R*), the highest being 0.206 observed between *Cd* and *b*/*B*.

It is important to note that the experimental datasets were collected from various hydraulic laboratories, each utilizing different methodologies^41,42^. As indicated in Table 1, the *Cd* varied significantly, ranging from 0.281 to 0.846. Additionally, the Froude number exhibited a range from 0.088 to 0.804. The reported variability in flow conditions indicates that the applied models in this study are designed to accommodate a wide spectrum of hydraulic scenarios, enhancing its generalizability.

## Method

In the current paper, ELM is used as a prediction model to estimate the *Cd* of rectangular sharp-crested side weirs. ELM has recently gained a strong reputation in the water resources field due to its fast learning, coverage, and good generalization capacity^43,44^, making it suitable for solving complex engineering problems. Additionally, the XGBoost model is chosen for its ability to handle feature interactions and its robustness against overfitting through boosting approaches^45,46^. Additionally, the LWLR is also included for its capability to adapt to local patterns in the data^47^, improving prediction accuracy in heterogeneous datasets. The proposed model, ELM-LWLR, combines the strengths of ELM, and LWLR models, potentially demonstrating strong performance in addressing complex hydraulic issues. Thus, the proposed model (ELM-LWLR) has been validated against robust models capable of effectively handling nonlinear problems, such as XGBoost, LWLR, and ELM, while also being compared with the standard model (MLR), which serves as the benchmark.

### Extreme learning machines (ELM)

ELM is an effective algorithm designed to train the Single Layer Feed Forward Neural Networks (SLFFNN), enhancing the training process and improving generalization capacity^48^. In the ELM model, the weights and bias values of the hidden layer are assigned randomly, which allows for significantly faster training and lower complexity compared to models trained using traditional backpropagation techniques. Only the output layer of the ELM is determined systematically, reducing the risk of unintended human interference.

To build an SLFFNN based on ELM principles, follow these steps: first, construct the network; second, randomly assign the weights and bias values; and third, compute the output weights by inverse the hidden layer output matrix. Assuming the dataset consists of *N* training samples organized by × matrix for input and output, the SLFFNN can be constructed with *L* hidden nodes, and its targets are described by the following equation:

1 $$\:\sum\:_{i=1}^{L}{\beta\:}_{i}G\left({wn}_{i}{x}_{j}\:+{b}_{j}\right)=\:{o}_{j}\:,\:\:\:\:j=\text{1,2},3,\dots\:N$$

where, G is the transfer function, $\:{\beta\:}_{i}$ is a vector representing the output weights values. Also, $\:{wn}_{i}$, and $\:{b}_{j}$ are the weight and bias values. Finally, term *x* is the input parameter(s). In this research, the log sigmoid transfer function is used, as shown below:

2 $$\:G=\:\frac{1}{1+{e}^{-x}}$$

To simplify the above equation, the term *H* represents the output results of hidden layer, denoted as $\:G\left({wn}_{i}{x}_{j}\:+{b}_{j}\right)$, and the predicted target vector which *T* includes the actual values (t_1_, t_2_,.t_N_), leading to the relationship $\:H\beta\:=\:T$ 

3 $$\:H=\:{\left(\begin{array}{ccc}G\left({wn}_{1}{x}_{1}\:+{b}_{1}\right)&\:\cdots\:&\:\left({wn}_{L}{x}_{1}\:+{b}_{L}\right)\\\:\vdots&\:\ddots\:&\:\vdots\\\:G\left({wn}_{1}{x}_{N}\:+{b}_{1}\right)&\:\cdots\:&\:G\left({wn}_{L}{x}_{L}\:+{b}_{L}\right)\end{array}\right)}_{N\times\:L}$$

And,

4 $$\:\beta\:=\:{\left[\begin{array}{c}{\beta\:}_{1}^{T}\\\:{\beta\:}_{2}^{T}\\\:.\:\\\:.\\\:.\\\:{\beta\:}_{L}^{T}\end{array}\right]}_{L\times\:M}\:\:\:,\:\:\:\:\:T=\:{\left[\begin{array}{c}{t}_{1}^{T}\\\:{t}_{2}^{T}\\\:.\:\\\:.\\\:.\\\:{ot}_{N}^{T}\end{array}\right]}_{N\times\:M}\:$$

The main aim of the ELM algorithm is to minimize the forecasting error $\:H\beta\:-\:T$. Thus, $\:\beta\:$ can be computed using Moore–Penrose generalized inverse:

5 $$\:H\beta\:=\:T$$

Then, $\:\beta\:$ can be linearly computed as follow:

6 $$\:\widehat{\beta\:}=\:{H}^{\dagger}\:T$$

where, $H^{\dagger}$ is Moore–Penrose generalized inverse matrix.

### XGBoost

XGBoost is a powerful boosting technique that combines multiple learning methods to achieve greater prediction accuracy than any single approach^49,50^. In recent years, it has gained widespread popularity across various fields, particularly in addressing challenges in the water resources sector. The fundamental concept of the XGBoost model primarily relies on an ensemble decision tree approach, which makes it commonly used in the field of data science. XGBoost creates predictions by using an internal method that combines the results from various individual trees. The primary goal of this model is to minimize the fitness function through the implementation of a gradient descent optimization strategy. Boosting-based approach is a powerful ensemble method that can combine thousands of lower-performing predictive models into a highly effective one. This is achieved by constantly integrating these models within acceptable parameter ranges, thereby enhancing overall performance. The objective function of the XGBoost can be written using Eq. 7.

7 $$\:{Obj}_{XGBoost}\left(\theta\:\right)=\:\sum\:_{i}L\left(\widehat{{y}_{i}},\:{y}_{i}\right)+\:\sum\:_{k}\varOmega\:\left({f}_{X}\right)$$

The objective function illustrated in the above equation includes a loss function represented by L and a regularization term $\:\varOmega\:\left({f}_{X}\right)$, which minimizes the variation in the output of the new tree. The terms $\:y$ and $\:\widehat{y}$ are the observed and predicted values.

An essential component of the XGBoost method is the utilization of a first-order approximation to optimize the objective function, employing an additive strategy. In each iteration, denoted as ‘t’, a new tree function ‘ft’ is added with the aim of minimizing the approximated objective (see Eq. 8)^51^. This process is a key part of how XGBoost operates, as it continually adds new learners to the model to progressively reduce the objective

8 $$\:L\left(t\right)\approx\:\sum\:_{i=1}^{n}l\left({y}_{i},{\widehat{y}}^{\left(t-1\right)}\:\right)+{g}_{i\:\:}{f}_{t}\left({x}_{i}\right)+\:\frac{1}{2}\:\:{h}_{i\:}{f}_{t}2\left({x}_{i}\right)+\:\varOmega\:\left({f}_{X}\right)$$

where *gi* and *hi* represent the statistics (first and second-order gradient) of the loss function. Detailed information about the XGBoost model can be found in the previous works^52^.

### Multiple linear regression (MLR)

MLR is a statistical model that specifies the independent variable, which in this study is *Cd*, using a linear equation system involving one or more input variables (*x*). The mathematical formula for the MLR model can be represented in the equation below:

9 $$\:Cd=\:{a}_{0}+\:\sum\:_{i=1}^{n}{a}_{i}{x}_{i}+e\:$$

The coefficients of MLR model are $\:{a}_{0}$, and $\:{a}_{i}$ can be computed using least square error method (LSM), the term e is prediction error, and the *n* is the total number of predictors.

The fitness function of this model can be represented in the following equations and should be minimized in order to determine the best fit-line for the measured *Cd* data sets.

10 $$\:Fitnss\:=\:\frac{1}{2 N}{\sum\:}_{i=1}^{N}{\left({Cd}_{obs\:i}-\:{Cd}_{pred\:i}\right)}^{2}$$

The N is the total number of the used *Cd* data samples, $\:{Cd}_{obs\:i}$, and $\:{Cd}_{pred\:i}$ are the measured and estimated *Cd* samples.

To find the optimal coefficients (i.e. $\:{a}_{0}$, $\:{a}_{1}$, $\:{a}_{3}$, etc.) of MLR model, the LSM is used as illustrated in the following equation^40^:

11 $$\:\widehat{a}\:=\:{\left({x}^{T}X\right)}^{-1}{x}^{T}\:Cd$$

### Locally weighted linear regression (LWLR)

LWLR is an improved version of classical MLR which used frequently in the areas of machine learning and statistics^40^. The LWLR model developed by researchers employs a lazy learning strategy with an instance-based approach^53^. Assume that Eq. 12 is used to specify the output projected given a set of predictors using a hypothesis $\:{y}_{\theta\:}\left(x\right)$^54^.

12 $$\:F=\:{y}_{\theta\:}\left(x\right)={\theta\:}^{T}x$$

In the above equation, the *x* is the input training data points, *θ* is the coefficients, and *F* is the output vector.

In the LWLR, the *θ* vector is can minimize the weighted square error between *x* and *F*. Since *w*_*i*_ is the weights assigned to the *i*^th^ training sample, Eq. 13 defines the fitness function^54^. It is important to mention that the fitness function is the mean square error.

13 $$\:Minimize\:G\left(\theta\:\right)=\:\sum\:_{i=1}^{n}{w}_{i}\:{\left({y}_{\theta\:}\left({x}_{i}\right)-{F}_{i}\right)}^{2}\:=\:{(x\theta\:-F)}^{T}\:w\left(x\theta\:-F\right)$$

where *F* is the output vector, *x* is the matrix containing the input training data, and *w* is a weighted diagonal matrix.

In order to minimize the fitness function of the LWLR (*G*(*θ*)), the differentiation of *G*(*θ*) in the above equation with respect to θ must be zero, as the following equation shows:

14 $$\:\frac{\partial\:F\left(\theta\:\right)}{\partial\:\theta\:}={x}^{T}wX\theta\:-{x}^{T}wy=0$$

Thus, the LWLR’s definition of *θ* is as follows:

15 $$\:\theta\:=\:{\left({x}^{T}wx\right)}^{-1}\:{x}^{T}\:wy$$

In the current paper, the weighted matrix in the LWLR model is found using a kernel function. As a result, Eq. (16) provides the weighted matrix that is used as the radial basis function.

16 $$\:{w}_{i,j}=exp\left(-\frac{{\left|{x}_{i}-{x}_{j}\:\right|}^{2}}{2\times\:{Tau}^{2}}\right)$$

The constant number $\:Tau$ in the formula above is one that the user defines. Also, it controls the spread of the radial basis kernel (RBF). Furthermore, RBF was chosen for its ability to capture nonlinear patterns^55–57^, emphasize neighboring data points, and produce smooth projections. Furthermore, its bandwidth parameter allows for effective modifications and has demonstrated strong performance in various regression tasks.

### Suggested model (ELM- LWLR)

The ELM model is defined as an appropriate number of neurons. The input weights and biases are randomly initialized. Rather than using a standard linear regression output layer, the ELM model implements the LWLR approach. This involves computing LWLR coefficients for each training data point based on local characteristics using a radial kernel function and an appropriate bandwidth parameter (see Eq. 17). Equation 6 for the ELM can be modified using Eqs. 13, 14, and 16 as follows:

17 $$\:\widehat{\beta\:}=\:{\left({H}^{T}wH\right)}^{-1}\:{H}^{T}\:wT$$

The radial kernel function is chosen to more effectively capture the non-linear relationships between the inputs and the target variable. During prediction, the ELM hidden layer outputs are computed for each test data point, and the corresponding *Cd* coefficients from the nearest training data points (based on the radial kernel function) are used to obtain the final ELM-LWLR model prediction.

**Fig. 1:**
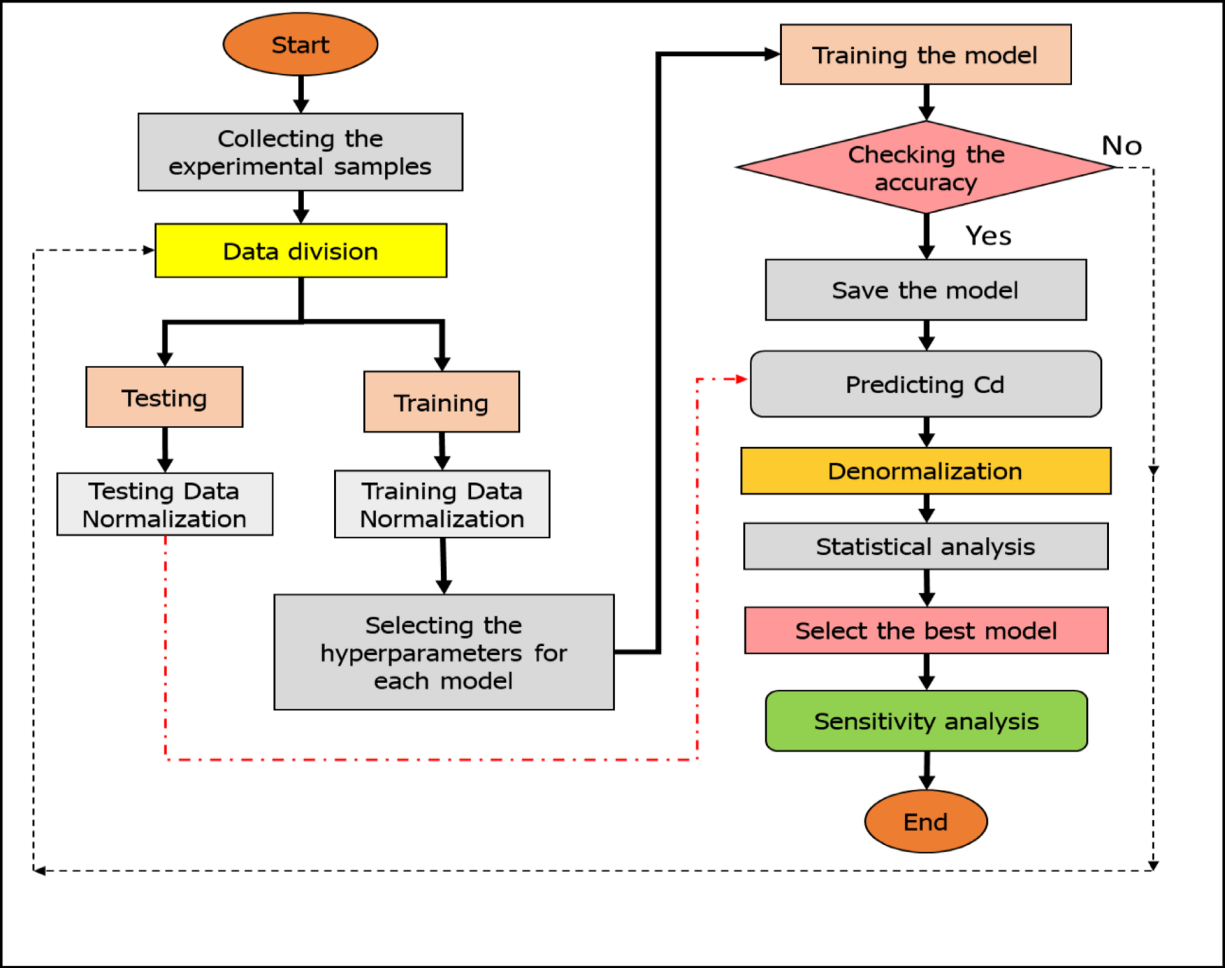
Flowchart showing the majors step for conducting prediction models.

The ELM-LWLR model was created using an experimental dataset of 162 samples, which included input variables and the target *Cd*. The comparable model and ELM-LWLR model were created using an experimental dataset of 162 samples, which included input variables and the target *Cd.* After collecting experimental samples, the data is preprocessed before being introduced to the prediction models. The preprocessing includes dividing the dataset into training and testing sets. The calibration step primarily depends on the training data, while the validation of the models’ performance relies on the testing data. The next step is normalization, which is crucial as it accelerates convergence and enhances model accuracy. Additionally, normalization ensures that features (inputs) contribute equally and ultimately reduces the influence of outliers.

The data was randomly divided into two sets; the testing data consisted of 49 samples, making up 30% of the total samples, while the remaining 70% was used for model construction. The data was then linearly normalized using a recommended procedure from a recent study to scale the data between zero and one^35^. The 70% allocation of the data for training the model provides a sufficient amount of information to effectively train the model^58^. Simultaneously, the remaining 30% of the data used for testing allows for ample data to evaluate the model’s ability to generalize well to new, unseen examples. Furthermore, the applied data division strategy strikes a balanced distribution between the training and testing sets, enabling accurate model training as well as a reliable assessment of the model’s performance.

The performance of the ELM-LWLR model was evaluated and compared to other models such as classical ELM, LWLR, XGBoost, and MLR. To accurately measure the potential improvements of the suggested model (ELM-LWLR), the structure of the model (i.e., hidden nodes) and the input layer weights, and bias values should be similar to the standard ELM model. This allows for a fair comparison between the two models to determine whether the proposed ELM-LWLR model achieves distinctive superiority. Notably, the hyperparameters of the ELM-LWLR model, such as the number of hidden neurons, the radial kernel function bandwidth parameter, and other relevant parameters, were assigned using trial and error methods. The trial-and-error method used in this paper for hyperparameter tuning which involves iteratively adjusting hyperparameters, training the model, and evaluating its performance. The adopted process continues until optimal performance of the applied prediction models is achieved. Finally, all models have been developed using Python, and the diagram in Fig. 1 is used to illustrate the key steps involved in conducting the prediction models in the current research.

### Statistical performances

There are seven statistical parameters that have been used to evaluate the performance of the applied models for predicting *Cd.* The metrics used are widely employed by researchers in assessing prediction models. The statistical parameters involved criteria used for observing the prediction error include root mean square error (*RMSE*), mean absolute error (*MAE*), uncertainty analysis (*U*_*95*_), and mean absolute percentage error (*MAPE*). Additional metrics used to evaluate the accuracy of the predictions include coefficient of determination (*R²*), Willmott’s index of agreement (*d*), and *R*.

The metrics used can be categorized into two sets. The first set quantifies the accuracy of the predicted values compared to the observed ones, including *R²*, *R*, and *d*. Ideally, these values should be equal to one. However, in practical applications, achieving these values is very difficult. Therefore, the best model produces values closer to one. The second set, consisting of *RMSE*, *MAPE*, *U*_*95*_, and *MAE*, measures overall prediction error, where ideal values are closer to zero. These indexes are preferred widely in previous works because they provide a comprehensive evaluation of model performance by assessing both accuracy and error magnitude. Using both the discussed sets of metrics may ensure a well-rounded assessment. Besides, the applied approach of assessing the models captures different properties of model performance rather than relying on a single evaluation criterion. In order to enhance the evaluation of the applied model, Combined Accuracy (*CA*) is used^59,60^. *CA* integrates key performance metrics (i.e., *RMSE*, *R²*, and *MAE*), providing an overall assessment of the prediction models, like the ideal point error^61^. Moreover, the percentage bias (*PBIAS*) is considered in this research as a significant statistical parameter that measures the overall tendency of a model’s predictions to be higher or lower than observed values. A positive *PBIAS* indicates underestimation (prediction results are lower than experimental ones), while a negative *PBIAS* indicates overestimation. A value of *PBIAS* close to zero signifies minimal bias. The mathematical expressions for these metrics can be found in the following equations (Eq. 18 to 26)^28,36^^62^,–^65^.

18 $$\:RMSE=\:\sqrt{\frac{1}{n}\sum\:_{i=1}^{n}{\left({Cd}_{{obs}_{i}}-{Cd}_{{pred}_{i}}\right)}^{2}\:}$$

19 $$\:MAE=\frac{\sum\:_{i=1}^{n}\left|{Cd}_{{obs}_{i}}-{Cd}_{{pred}_{i}}\right|}{n}$$

20 $$\:d=1-\frac{\sum\:_{i=1}^{n}({Cd}_{{obs}_{i}}-{Cd}_{{pred}_{i}}{)}^{2}}{\sum\:_{i=1}^{n}(\left|{Cd}_{{pred}_{i}}-\:\stackrel{-}{{Cd}_{obs}}\right|{+\:\:\left|{Cd}_{{obs}_{i}}-\:\stackrel{-}{{Cd}_{obs}}\right|)}^{2}\:\:}$$

21 $$\:{R}^{2}=1-\frac{\sum\:_{i=1}^{n}({Cd}_{{obs}_{i}}-{Cd}_{{pred}_{i}}{)}^{2}\:}{\sum\:_{i=1}^{n}{\left({Cd}_{{pred}_{i}}-\stackrel{-}{{Cd}_{pred}}\right)}^{2}}$$

22 $$\:MAPE\text{\%}\:\:=100\times\:\frac{1}{n}\sum\:_{i\:=\:1}^{n}\frac{{Cd}_{{obs}_{i}}-{Cd}_{{pred}_{i}}}{{Cd}_{{obs}_{i}}}$$

23 $$\:R=\frac{\sum\:_{i=1}^{n}\left[\left({Cd}_{{obs}_{i}}-\stackrel{-}{{Cd}_{obs}}\right)\left({Cd}_{{pred}_{i}}-\stackrel{-}{{Cd}_{pred}}\right)\right]}{\sqrt{\sum\:_{i=1}^{n}{\left({Cd}_{{obs}_{i}}-\stackrel{-}{{Cd}_{obs}}\right)}^{2}\sum\:_{i=1}^{n}{\left({Cd}_{{pred}_{i}}-\stackrel{-}{{Cd}_{pred}}\right)}^{2}\:\:\:\:}\:\:\:}$$

24 $$\:{U}_{95}\:=\:\frac{1.96}{n}\sqrt{\sum\:_{i=1}^{n}{\left({Cd}_{{obs}_{i}}-\stackrel{-}{{Cd}_{obs}}\right)}^{2}\:+\sum\:_{i=1}^{n}({Cd}_{{obs}_{i}}-Cd{)}^{2}\:}\:$$

25 $$\:CA=0.33\times\:\left(MAE+RMSE+\left(1-{R}^{2}\right)\right)$$

26 $$\:PBIAS=\:\frac{\sum\:_{i=1}^{n}\left({Cd}_{{obs}_{i}}-{Cd}_{{pred}_{i}}\:\right)}{\sum\:_{i=1}^{n}{Cd}_{{obs}_{i}}}\times\:100\%$$

where for this study, $\:{Cd}_{{obs}_{i}}$, $\:{Cd}_{{pred}_{i}}$ are the observed *Cd* at *i*^*th*^ data point and predicted (Cd), *n* is the total data points. The means of observed and predicted *Cd* values are $\:\stackrel{-}{{Cd}_{obs}}$, $\:\stackrel{-}{{Cd}_{pred}}$, respectively.

## Model accuracy in training and testing

### Modeling training results

This section of the paper discusses the capability of the applied models in predicting the *Cd* coefficient. It is important to note that 70% of the samples were used for training the models, and the remaining 30% were used for the testing phase for validation purposes. The performance of the four prediction models during the calibration phases is assessed using several statistical metrics, and the results are summarized in Table 2. According to the given results, it is clear to observe that the AI-based models have superior performance over the classical MLR model, which produces much lower performance (*RMSE* = 0.069, *MAE* = 0.053, *R* = 0.66). This means that the MLR, which uses a linear function, is unable to simulate the underlying nonlinear relationship between *Cd* and the input hydraulic variables. However, the ELM-LWLR, LWLR, and XGBoost models provide excellent performance in the training phase, with *RMSE* ranging from 0.011 to 0.016, *MAE* from 0.008 to 0.009, and *R* from 0.985 to 0.994.

The ELM model has a fair performance, which is better than MLR but has lower accuracy than the other AI models and LWLE. Moreover, models such as MLR, ELM, and ELM-LWLR exhibit lower *PBIAS* values, indicating minimal bias. Among them, ELM-LWLR shows the least bias (*PBIAS* = −0.03), followed by MLR (*PBIAS* = −0.005%) and ELM (*PBIAS* = 0.052%). Also, the XGBoost model exhibits moderate bias, while the classical LWLR presents the highest deviation, with *PBIAS* of 0.173%.

As the training phase requires providing the models with both input and output values to train the model and conduct the calibrations, it is crucial to examine the performance of each model during the testing phase, where the model receives only the input, providing a more comprehensive understanding of the model’s performance. It is important to note that all the hyperparameters of the models are provided in Table S1 in the supplementary files.

In this study, the aim is to examine how the LWLR approach enhances the performance of the ELM model. The structures of both the ELM and ELM-LWLR models, as well as the hidden layer parameters (weights and biases), are kept consistent. Trial-and-error methods were employed to select the hyperparameters of the models. According to Table S1, the ELM-LWLR model parameters are set with *Tau* at 0.1 and a hidden layer of 20 nodes. With these parameters, the model achieves a lower *RMSE* of 0.016. Furthermore, the model is significantly sensitive to changes in the *Tau* value. When Tau is adjusted to 0.15, the prediction accuracy decreases, resulting in an *RMSE* of 0.0195. Similarly, when *Tau* is set to 0.8, the *RMSE* increases to 0.25.

In order to maintain consistent training conditions and evaluate how LWLR improves the ELM-LWLR model, the same *Tau* values were used for training the LWLR model. It was observed that the LWLR model is also sensitive to the Tau parameter. With Tau values of 0.15 and 0.8, the *RMSE* values are 0.22 and 0.30, respectively, compared to 0.016 at *Tau* 0.1. Additionally, three values of hidden nodes, namely 10, 20, and 25, were tested, revealing that 20 nodes yield the best performance for both ELM and ELM-LWLR models. The results during the training phase show slight variations. For instance, the RMSE values for both models are 0.185 and 0.43, respectively, with 10 hidden nodes. With 20 hidden nodes, the *RMSE* values for both models are slightly improved to 0.18 and 0.415, respectively. Thus, it can be concluded that using 20 hidden nodes leads to more stable models with lower *RMSE*, as reported in Table 2.

When selecting hyperparameters for XGBoost, the rationale behind each choice significantly impacts model performance. The learning rate influences convergence speed. Lower values allow for more stable learning but may require additional boosting rounds. This paper tested several learning rates, including 0.10, 0.15, and 0.20, yielding *RMSE* values of 0.025, 0.13, and 0.024, respectively. Notably, at a learning rate of 0.15, the model performs well. However, when testing a learning rate of 0.16, a slight improvement is observed, resulting in an *RMSE* of 0.011. Additionally, the model’s performance is affected by the maximum depth. This essential parameter was set within the range of 5 to 10, with XGBoost showing the best results at a maximum depth of 7.

**Table 2:** Assessment of the performance of the predictive modeling approaches during the training phase.

Models	*RMSE*	*MAE*	*PBIAS* (%)	*d*	*R*
MLR	0.069	0.053	0.005	0.783	0.663
ELM	0.039	0.031	0.007	0.948	0.904
XGBoost	0.011	0.008	0.052	0.997	0.994
LWLR	0.016	0.009	0.173	0.992	0.985
ELM-LWLR	0.016	0.009	−0.003	0.992	0.985

### Modeling testing results

Table 3 summarizes the test statistics of the applied models in predicting the *Cd* coefficient using testing data. From the results reported, it is clear that the proposed ELM-LWLR model has a distinct advantage over the other models in terms of accuracy and performance. The ELM-LWLR model has the highest accuracy performance with *R*, and *d* at 0.968 and 0.9844, respectively, and it generates very low forecasting errors (*RMSE* = 0.027, *MAE* = 0.018). The second-best model is XGBoost, with *RMSE* = 0.038, *MAE* = 0.027, *d* = 0.963, and *R* = 0.925. According to similar statistics, LWLR is the third-best model, while ELM is the fourth best, and MLR has the lowest performance in this study. Furthermore, the superiority of ELM-LWLR over the other models can be quantified in terms of *RMSE* reduction. As a result, the predictive performance has been significantly enhanced, with improvements of 37.21%, 28.95%, 48.08%, and 64.94% compared to LWLR, XGBoost, ELM, and MLR, respectively. These metrics show that the ELM-LWLR model is better at minimizing prediction errors and adapting to the underlying patterns in the *Cd* data. Also, the finding highlights the effectiveness of the ELM-LWLR model in capturing complex local data patterns and refining linear computations within the ELM framework. Furthermore, the substantial accuracy boost across diverse benchmarks underscores the model’s robustness and adaptability, making it a superior choice for addressing intricate predictive challenges.

**Table 3:** Assessment of the performance of the predictive modeling approaches during the testing phase.

Models	*RMSE*	*MAE*	*PBIAS* (%)	*d*	*R*
MLR	0.077	0.060	−0.157	0.774	0.713
ELM	0.052	0.039	−0.546	0.927	0.875
XGBoost	0.038	0.027	1.484	0.963	0.936
LWLR	0.043	0.024	1.565	0.949	0.925
ELM-LWLR	0.027	0.018	−0.130	0.984	0.968

The *PBIAS* values indicate the degree to which each model tends to either overestimate or underestimate the numbers that really exist. While ELM (−0.546) and MLR (−0.157) both have a modest tendency to overstate, ELM-LWLR (−0.130) has the least amount of bias among the three methodologies. On the other hand, XGBoost (1.484) and LWLR (1.565) greatly underestimate the values, which refers to the fact that their predictions are often lower than the actual data.

The Wilcoxon Signed-Rank Test (WSRT) was performed to evaluate the absolute differences in errors between the proposed ELM-LWLR model and comparable models. The WSRT approach is selected in the current research due to its capability to handle non-normally distributed data. Table 4 displays the results, encompassing the mean difference, 95% confidence interval (calculated using bootstrap method with 100000 iterations), and *p-values*. The results show that the ELM-LWLR model illustrates a statistically significant difference (*p* < 0.05) compared to other predicting models. Also, the attain results underscore that the suggested model has a more enhanced performance than others. Furthermore, the mean difference indicates a significant decrease in absolute error, with the lower and higher limits of the 95% confidence range further substantiating this reliability. The results validate that the ELM-LWLR model surpasses competing models regarding prediction error reduction.

**Table 4:** Wilcoxon Signed-Rank test comparing the LWELM model with other models.

Comparison models	Mean differences	Lower Bounds	Upper Bounds	*p*-value	Statistically significance
LWELM vs. MLR	0.0412	0.0274	0.0555	1.439$\:\times\:{10}^{-6}$	Yes
LWELM vs. ELM	0.0212	0.0091	0.0332	0.0044	Yes
LWELM vs. XGBoost	0.0323	0.0188	0.0463	0.0003	Yes
LWELM vs. LWLR	0.0362	0.0243	0.0489	8.270$\:\times\:{10}^{-8}$	Yes

The analysis of time complexity and computational scalability highlights the running times for each model using all input features, The current paper incorporates all input features to ensure optimal performance and thoroughly evaluate each model’s complexity. As the number of input features increases, model complexity and convergence time also grow, reflecting the effort required to achieve optimal results. The models used in this work were executed on a laptop running a 64-bit operating system (Windows 10) with Python 3.11, an Intel(R) Core (TM) i7-4600U CPU 2.10 GHz (2.70 GHz), 4.00 GB of RAM, and a 250 GB SSD hard drive. Notably, The Python programming language was used to develop the proposed models, and the corresponding code is provided in Appendix K of the supplementary file. According to Table 5, the XGBoost model required the longest training time at 0.3589 s compared to other used models. However, the other models (MLR, LWLR, ELM, and ELM-LWLR) demonstrated significantly shorter times of 0.0137 s, 0.0165 s, 0.0162 s, and 0.0181 s, respectively.

**Table 5:** Analyzing train time and complexity across different models.

Model	Runing time per second (s)
XGBoost	0.3589
MLR	0.0137
LWLR	0.0165
ELM	0.0162
ELM-LWLR	0.0181

It is vital to note that the ELM-LWLR model leverages the strengths of both ELM and LWLR which aims to improve accuracy of *Cd* prediction without sacrificing computational efficiency. Also, the used ELM model employs a single hidden layer for fast training, while LWLR introduces localized adaptation, enabling responsiveness to variations in data distributions. The ELM-LWLR model shows a slight increase in training time compared to ELM and LWLR which reflects the complexity of integrating the two algorithms. Furthermore, the integration of LWLR and ELM allows the ELM-LWLR model to effectively capture intricate patterns in the *Cd* data while maintaining relatively low computational demands. Despite its slightly higher complexity, ELM-LWLR is considered as the most accurate prediction model. Overall, while XGBoost exhibits robust performance, ELM-LWLR stands out as a strong alternative for scenarios requiring a balance between speed and adaptability, particularly with complex datasets. Besides, the proposed ELM-LWLR model demonstrates significant potential for practical applications where quick turnaround times are critical.

**Fig. 2:**
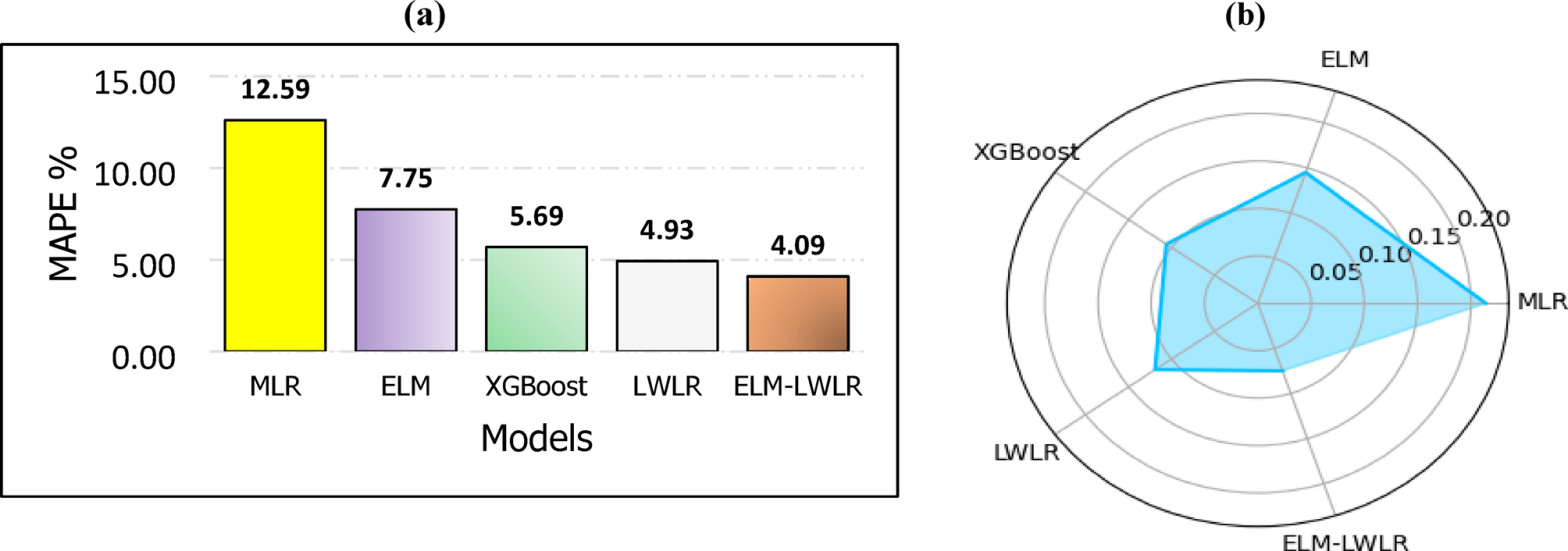
Model Comparison. (**a**) *MAPE*%, and (**b**) *U*_*95*_.

Furthermore, the ELM-LWLR model provides the lowest values of *MAPE* as seen in Fig. 2a, with *MAPE* of 4.09%. Also, the ELM-LWLR is followed by LWLR (*MAPE* = 7.75%), XGBoost (*MAPE* = 5.69%), ELM (*MAPE* = 7.75%), and MLR (*MAPE* = 12.59%), respectively. Additionally, according to Fig. 2b, the suggested ELM-LWLR model reported the lowest value of uncertainty analysis (*U*_*95*_ = 0.075), followed by XGBoost (0.106), LWLR (0.119), ELM (0.145), and MLR (0.214), respectively. The reported results show the superior predictive accuracy of the ELM-LWLR model compared to other applied models. The consistently lower *MAPE%* indicates that the ELM-LWLR model not only performs well but also provides a more reliable prediction capability. Furthermore, the findings from Fig. 2b highlight the ELM-LWLR model’s more effectiveness in minimizing uncertainty value (*U*_*95*_) than XGBoost, ELM, MLR, and LWLR models. The attained findings suggest that the ELM-LWLR model not only achieves better accuracy but also offers greater confidence in its predictions.

**Fig. 3:**
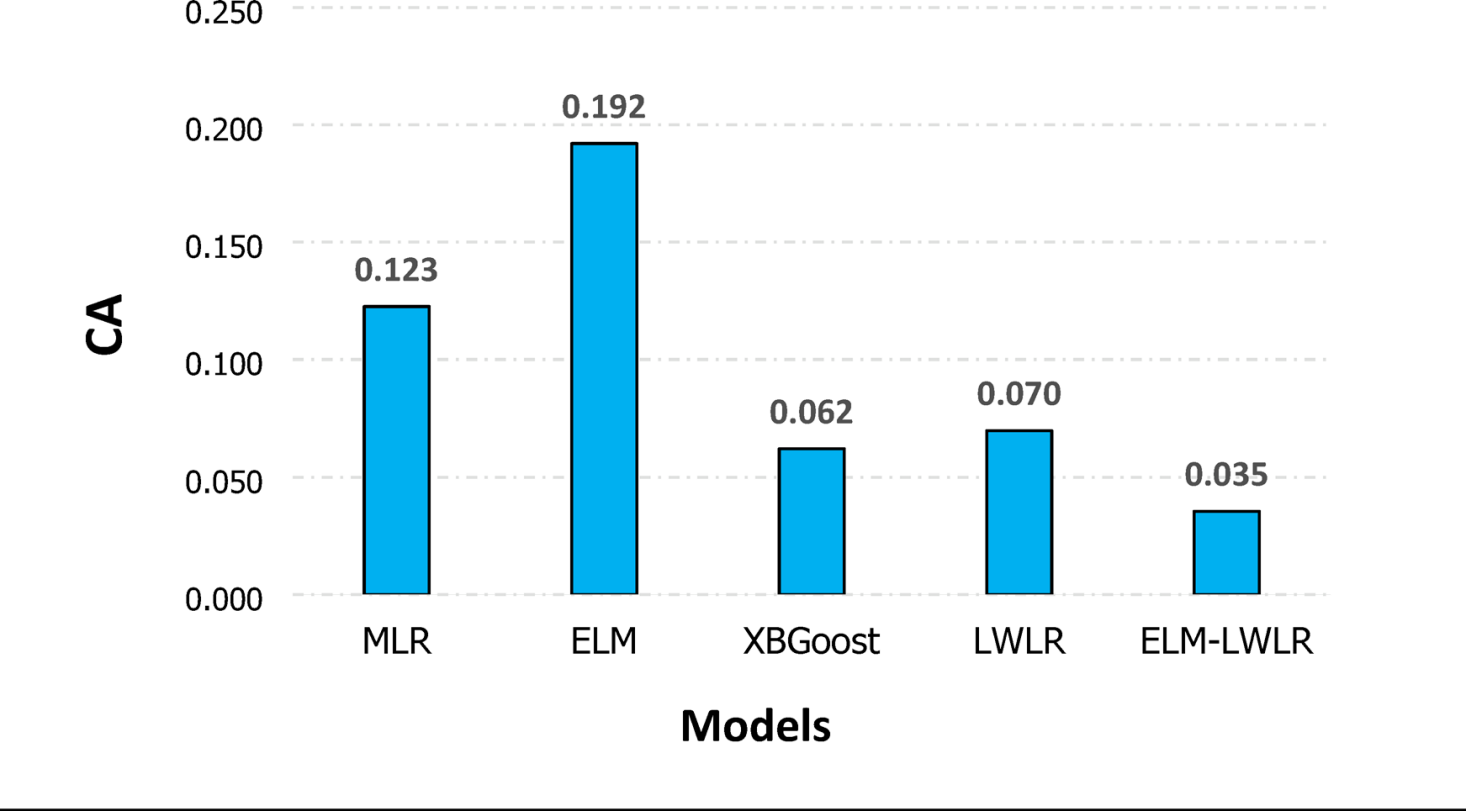
Comparison of model prediction accuracy using *CA*.

To assess prediction models more effectively, *CA* has been calculated for each model. Figure 3 shows that every model exhibited varied degree of accuracy in the prediction of *Cd.* The ELM-LWLR among the employed models have the best prediction accuracy, achieving the lowest *CA* of 0.035. The results confirm that the adopted model has a robust prediction and generalizing capacity. Nevertheless, the ELM and MLR models produced the highest *CA* of 0.192, and 0.132, respectively, suggesting that they have a significant limitation in capturing the complicated patterns of *Cd* data. Furthermore, the other models are XGBoost and LWLR models exhibit a modest prediction performance with *CA* values of 0.062 and 0.07 respectively.

**Fig. 4:**
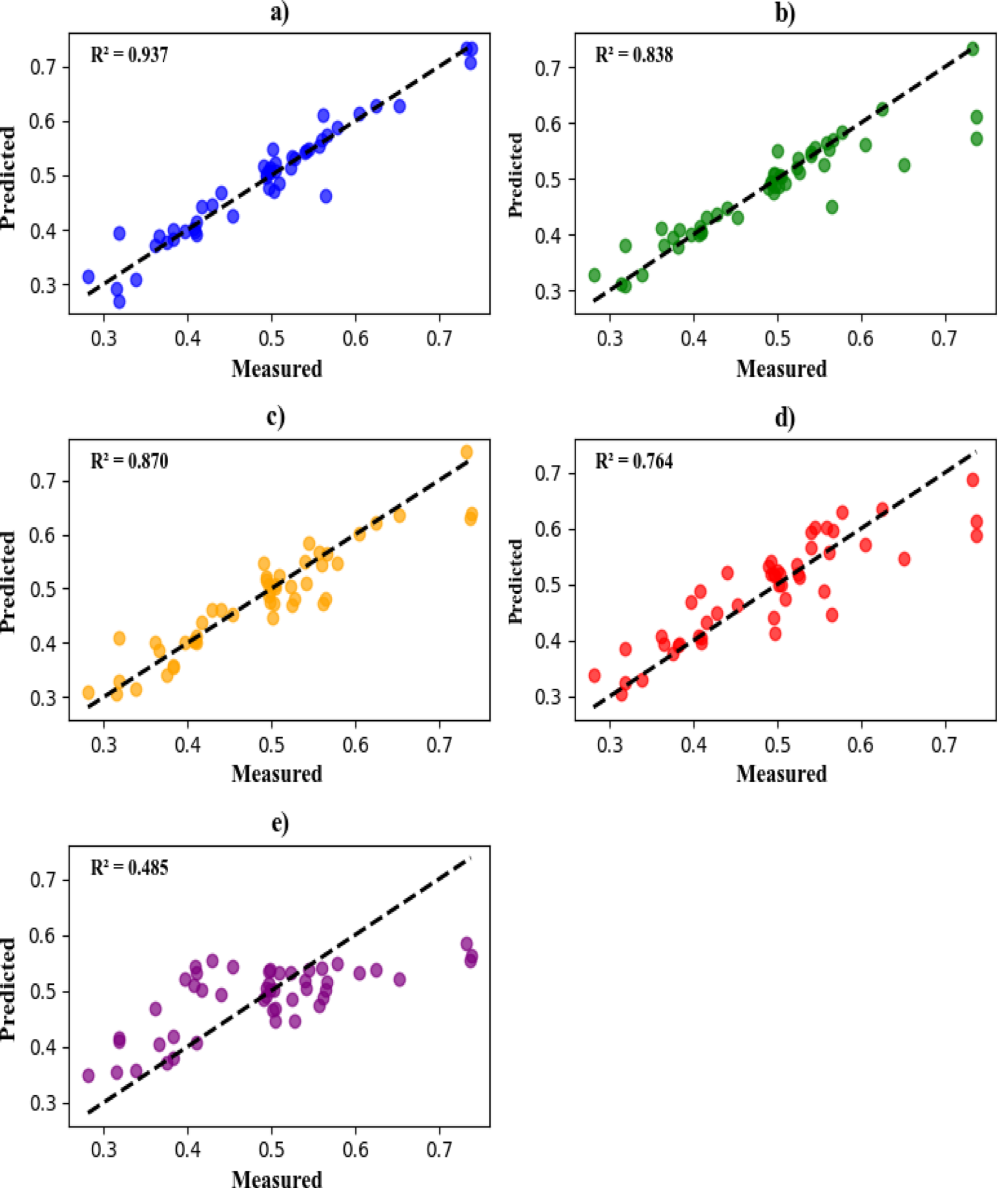
Scatter plots for assessing the models. Panels a, b, c, d, and e are representing ELM-LWLR, LWLR, XGBoost, ELM, and MLR, respectively.

Figure 4(a-e) shows the scatter plot diagrams, which are crucial as they visually diagnose how the individual prediction points align relative to the experimental reference values. These figures have been created using the testing data set, providing more information on the deviation of prediction points from the 1:1 ideal line. It can be observed that all the models except ELM-LWLR generated prediction points that deviate significantly from the laboratory *Cd* values. This indicates a substantial divergence between the ideal 1:1 line and the simulated line, an observation that can be seen in all models except ELM-LWLR. According to Fig. 4 (a to e), the ELM-LWLR model has the highest *R*-squared value of 0.937, followed by XGBoost (*R*^*2*^ = 0.870), LWLR (*R*^*2*^ = 0.838), ELM (*R*^*2*^ = 0.764), and MLR (*R*^*2*^ = 0. 485).

**Fig. 5:**
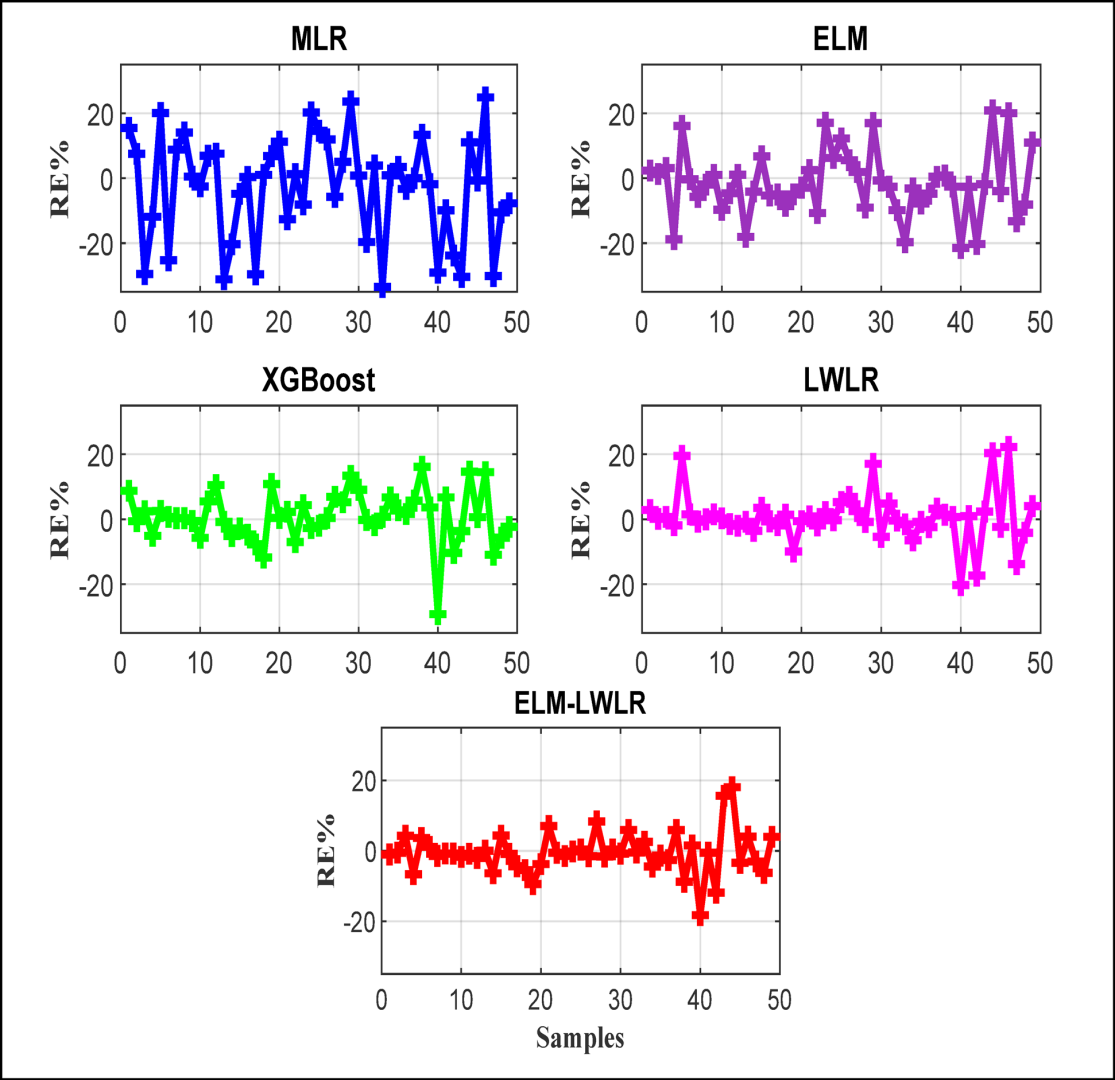
Relative error of the comparative models.

Relative error percentage (*RE%*) is very important to assess the qualifications of each model separately, as it provides insights into the accuracy of the applied models for predicting *Cd.* The RE diagram, as shown in Fig. 5, highlights the variability in model performance across different values of the *Cd* coefficient. According to the figure, the ELM-LWLR model consistently exhibited lower relative errors compared to other models, suggesting its superior performance in predicting *Cd.* The model has very low *RE*, and most of the predicted points have *RE* ranges from − 10 to 10%. Also, approximately 3 data points have a relative error close to 20%, while the other comparable models generated several points having relative errors exceeding 20%. The results also depict that the MLR struggles in providing a fair prediction, while the XGBoost and LWLR models have almost similar performance.

**Fig. 6:**
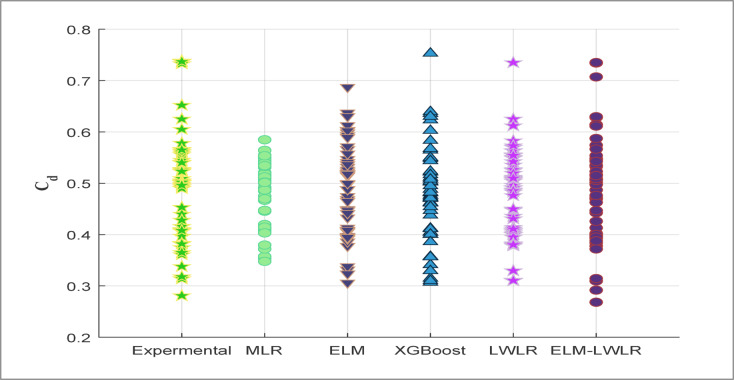
Dot plots showing a comparison between experimental *Cd* and predicted values.

**Fig. 7:**
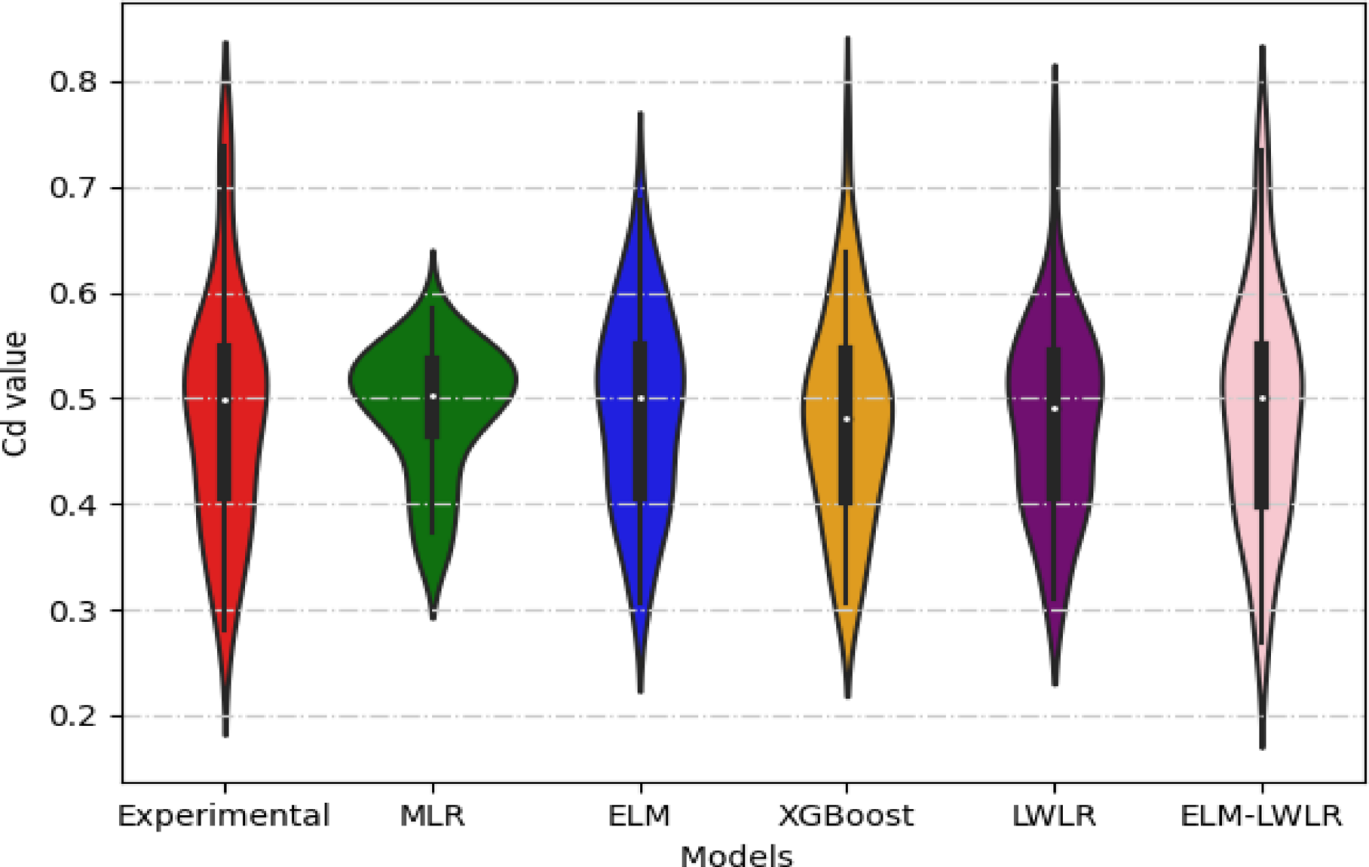
Violin plots showing a comparison between experimental Cd and predicted values.

To better understand the relationship between the experimental *Cd* and the predicted values, Dotplot and Violin diagrams, as seen in Figs. 6 and 7, have been created using the testing dataset. They are essential to visualize the distribution and provide a comprehensive comparison between the predicted *Cd* and reference experimental *Cd* values. The dot plot presented in Fig. 6 illustrates the experimental *Cd* values versus various models, including MLR, ELM, XGBoost, LWLR, and ELM-LWLR. This visualization offers a clear comparative analysis of the models’ performance across different ranges of *Cd* values, highlighting their reliability and effectiveness in predicting *Cd.* The figure indicates that the ELM-LWLR model exhibits outstanding predictive accuracy, with its predicted values closely aligning with the experimental results. Furthermore, the model demonstrates superior performance compared to other models, particularly in accurately predicting extreme values of *Cd.* According to Fig. 6, the ELM and MLR models could not adequately mimic the highest and lowest values of *Cd*, and their predictions were not as good as the ELM-LELR, XGBoost, and LWLR models. Also, when it comes to the lowest *Cd* values (below 0.3), only the ELM-LELR model provides accurate predictions among the comparable models.

The Violin Plot in Fig. 7 shows the distribution of *Cd* samples predicted through the models proposed in this study, comparing them with the experimental *Cd* values. It also displays important statistical values, such as the median and interquartile range (IQR). The white circle in the middle of each figure represents the median, and the black rectangle represents the IQR. It can be noted that the proposed ELM-LELR model has almost the same distribution as the laboratory-measured *Cd* values and is almost identical to it in terms of median and IQR values, indicating its superiority over the other models, which showed somewhat fluctuating performance.

**Fig. 8:**
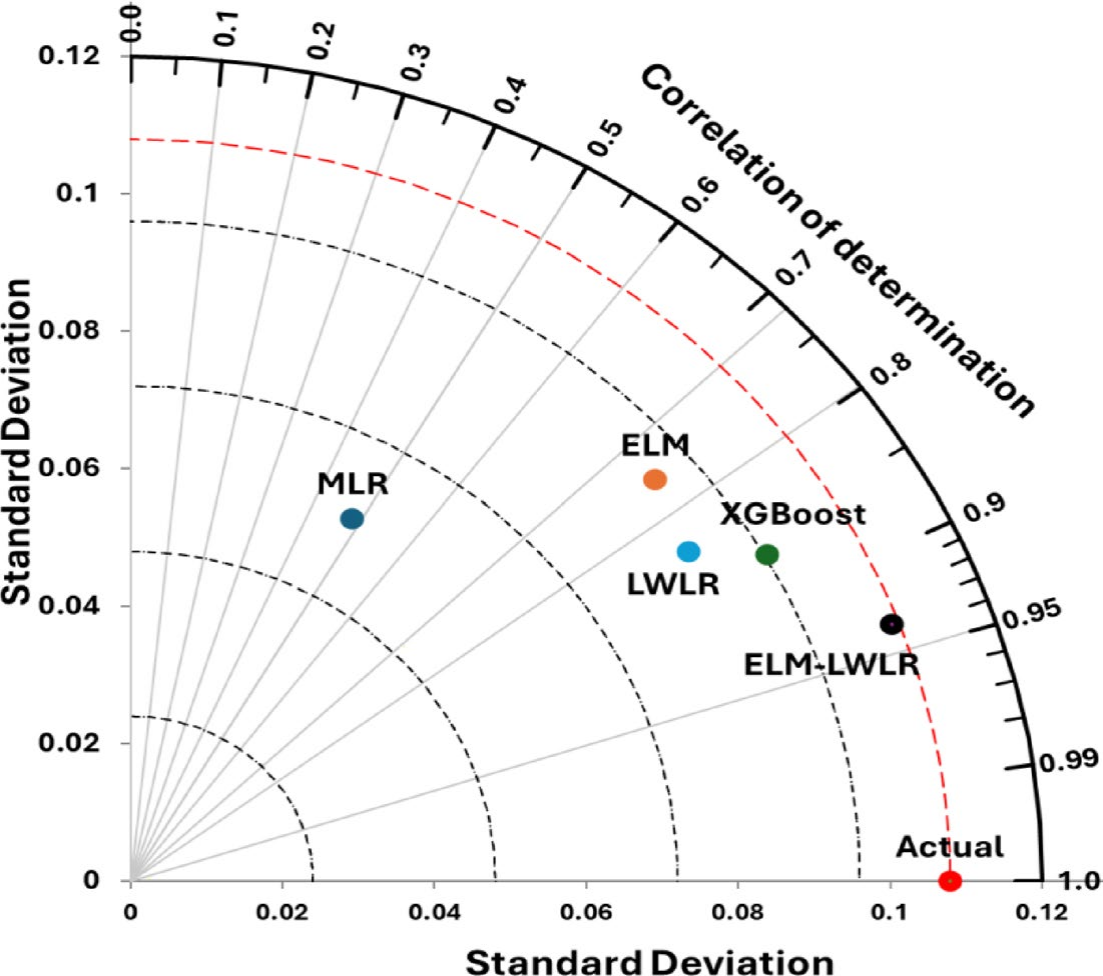
Taylor diagram showcasing the similarity between experimental *Cd* values and predictions achieved by various models.

The Taylor diagram as presented in Fig. 8 is a powerful tool for evaluating and comparing the performance of different predictive models against experimental *Cd.* The figure summarizes several key statistical metrics, such as the *R²* and standard deviation. Visually, the ELM-LWR model appears closest to the experimental *Cd*, indicating higher predictive accuracy than the others. Moreover, other models (XGBoost and LWLR) demonstrate good predictive accuracy, although they are relatively farther from the experimental values. In contrast, ELM and MLR are shown to be much farther from the experimental values, reflecting their poor capability in modeling *Cd* and resulting in lower *R²* values and standard deviations compared to the actual *Cd.* The visualization plot demonstrate that the model is effectively capturing the relationship between input features and *Cd*. Overall, the improved performance for ELM-LWLR model can be attributed to the hybrid nature of the ELM-LWLR approach, which effectively combines the global learning capabilities of ELM with the local adaptation of LWLR. These features of the hybrid model allow it to generalize well while also capturing local variations in the data.

**Fig. 9:**
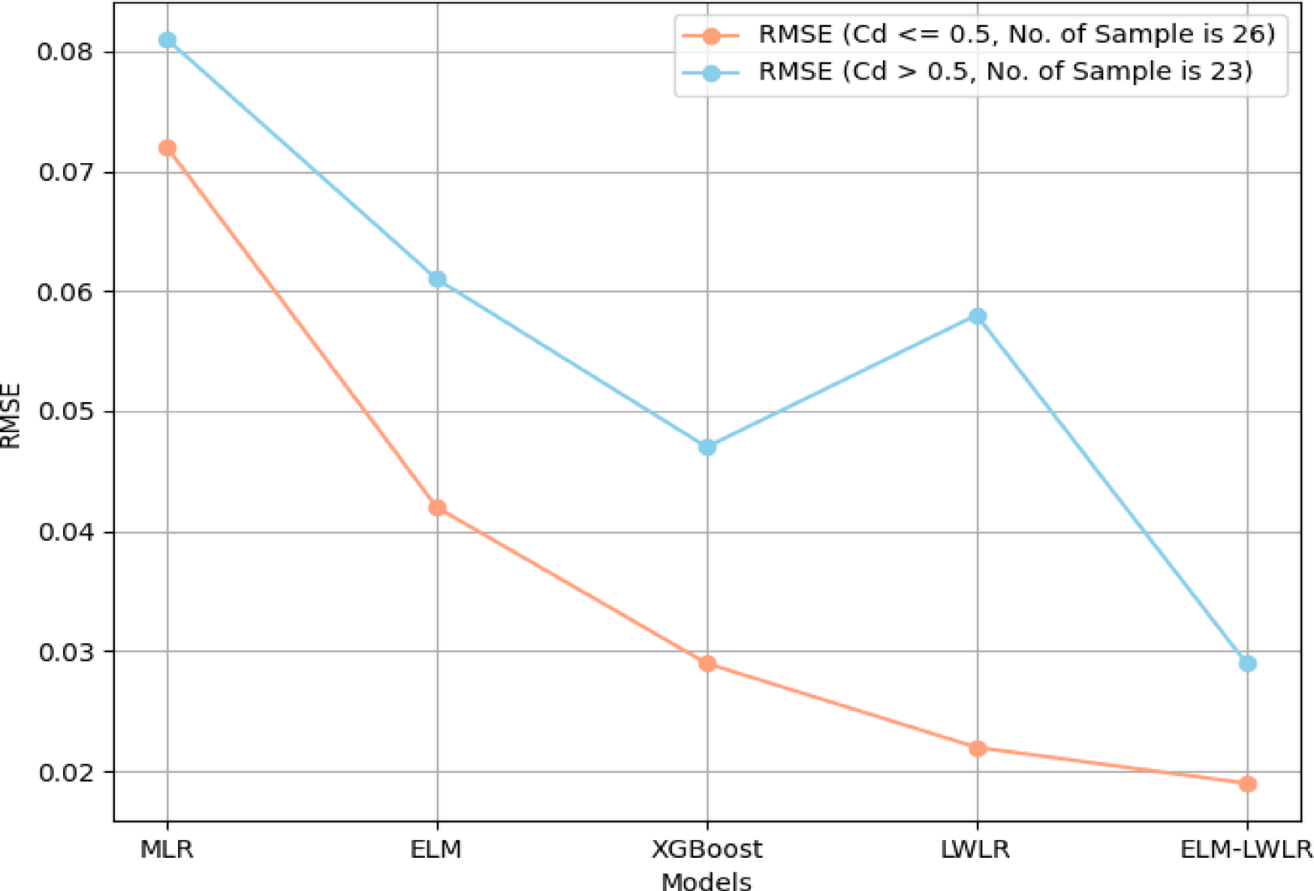
Comparison of *RMSE* for Different Models.

As previously discussed, some models struggled to accurately predict peak *Cd* values. To analyze model performance in predicting extreme values (lowest and highest Cd), the experimental *Cd* data was divided into two sets: one with *Cd* ≤ 0.5 (26 samples) and another with *Cd* > 0.5 (23 samples). Both sets are considered representative due to their similar sample sizes. The performance of each model was evaluated using *RMSE*. Figure 9 reveals that MLR, ELM, and XGBoost performed poorly in predicting the *Cd* ≤ 0.5 group, while ELM-LWLR emerged as the best model with an *RMSE* below 0.02. For the *Cd* > 0.5 group, ELM-LWLR again achieved the lowest *RMSE* (below 0.3). Accordingly, it can be concluded that the ideal model should have consistent performance across the two *Cd* data groups, minimizing the difference in *RMSE*. This is because a model that performs well on one group but poorly on the other would not be as reliable or generalizable as a model that maintains similar accuracy across the different data distributions. Accordingly, the ideal model should be able to adapt to the varying characteristics of the data, rather than being overly specialized for one specific data range. The reported results showed that the ELM-LWLR stands out in this regard, exhibiting minimal variation in *RMSE* between the two groups. Overall, the ELM-LWLR is the best model as it handles the different data patterns and characteristics, making it a more robust and versatile model compared to the other approaches.

### Sensitivity analysis

**Fig. 10:**
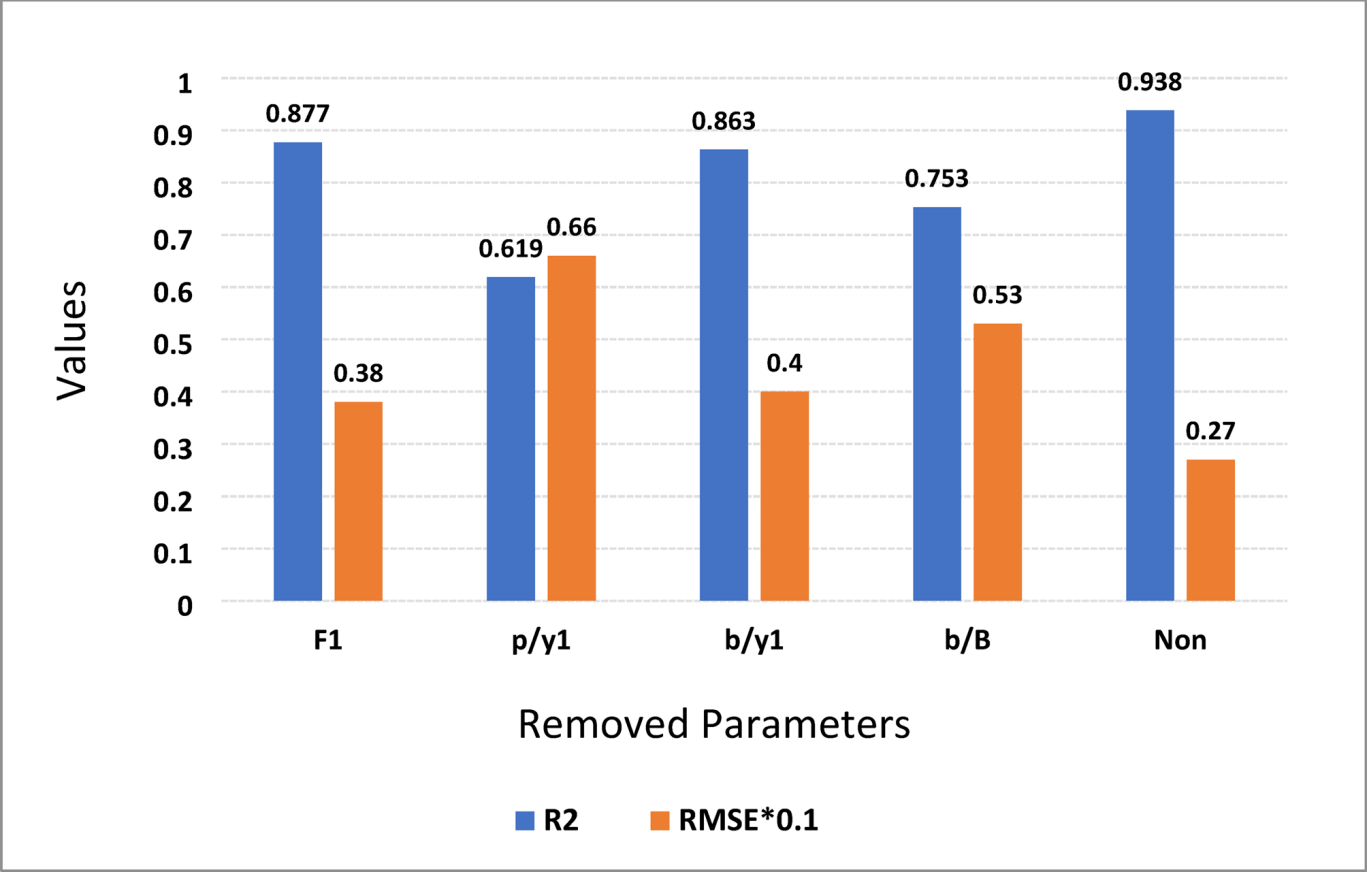
Sensitivity investigation using the ELM-LWLR model for the testing stage.

Sensitivity analysis (SA) plays a crucial role in determining which input parameter most significantly impacts the prediction of *Cd* coefficient, utilizing the top-performing ELM-LWLR model. SA employs various combinations of the testing dataset to pinpoint critical input parameters. As detailed in Table 6; Fig. 10, different input variations are generated by systematically excluding one variable at a time in each trial, allowing for the assessment of its effect on bearing capacity outcomes. The influence of each input parameter on the output (*Cd*) is evaluated using metrics such as *RMSE* and *R*^*2*^. The findings presented in Fig. 10 indicate that the removal of the input parameters such as *p*/*y*1, and *b*/*B*, results in the most significant changes, evidenced by a decrease in *R*^*2*^ and an increase in *RMSE* (*R*^*2*^ = 0.619, and 0.753, *RMSE* = 0.066, and 0.053), more than other parameter combinations. The analysis reveals that *p*/*y*1, and *b*/*B* are the primary factors affecting the estimation of *Cd* coefficient. This conclusion is consistent with earlier experimental research that utilized SA and found that *b*/*B* is an impactful parameter^7^.

**Table 6:** Input combination used for sensitivity analysis.

Remove Element	Input combination
*F1*	*Cd* = *f (p*/*y1*,* b*/*y1*,* b*/*B)*
*p*/*y*1	*Cd* = *f (F1*,* b*/*y1*,* b*/*B)*
b/y1	*Cd* = *f (F1*, *p*/*y1*,* b*/*B)*
*b*/*B*	*Cd* = *f (F1*, *p*/*y1*,* b*/*y1)*
Non	*Cd* = *f (F1*, *p*/*y1*,* b*/*y1*,* b*/*B)*

### Validating the proposed model against several forecasting models developed in previous work

The proposed ELM-LWLR model has been validated against several benchmark models and empirical equations developed in previous studies. Specifically, the first ten models and empirical equations used by hydraulic researchers^7^ with the same data are listed in Table 7. The researchers employed various machine learning models, including the Group Method of Data Handling (GMDH) and feedforward neural network (FFNN), as well as eight empirical equations. The results showed that the GMDH model outperformed the FFNN and other empirical equations, achieving a lower *RMSE* of 0.038 and a higher *R*-value of 0.779. Additionally, a recent study utilized a novel model called Extra Tree Regression (ETR), a modified version of RF model, and the RF model. The ETR model demonstrated better performance with an *R*-value of approximately 0.871^8^. Furthermore, a hybrid model has been developed based on KELM and GWA to predict the *Cd* and the model provide a relatively good prediction (*R*^*2*^ = 0.859)^23^.

Other researchers developed a hybrid model by integrating the ANFIS with several metaheuristic algorithms, including GA, Differential Evolution (DE), and PSO^66^. The study found that the ANFIS-DE model achieved the highest accuracy, with *R²* = 0.859 and *RMSE* = 0.077. Furthermore, a hybrid model was developed by combining SVR with Invasive Weed Optimization (IWO) to “predict the discharge coefficient of a stepped morning glory spillway”^67^. The SVR-IWO model was validated against classical SVR, Kernel Ridge Regression (KRR), and GPR. The study found that the hybrid model demonstrated superior performance, achieving a higher *R²* (0.646) and a lower *RMSE* (0.131). Based on the reported models and empirical equations used for predicting the *Cd*, the proposed model in the current study has shown a significant improvement, achieving the highest *R*-value of 0.938, which is higher than the other models.

**Table 7:** Evaluating the proposed model with existing models and empirical equations.

Reference	Model/Equation	*RMSE*	*R* ^2^
^7^	Ebtehaj et al.	0.038	0.779
^68^	Nandesamoorthy and Thomson	0.071	0.434
^69^	Yu-Tech	0.062	0.452
^70^	Ranga Raju et al.	0.072	0.453
^71^	Hager	0.118	0.438
^72^	Singh et al.	0.127	0.351
^73^	Jalili and Borghei	0.146	0.116
^41^	Emiroglu et al.	0.166	0.409
^7^	FFNN	0.063	0.605
^7^	GMDH	0.038	0.779
^23^	KELM-GWA	0.018	0.859
^67^	SVR-IWO	0.131	0.646
^66^	ANFIS-DE	0.077	0.872
^8^	ETR	0.0208	0.922
	RF	0.027	0.871
**Proposed model**	**ELM-LWLR**	**0.027**	**0.938**

## Discussion

Modeling *Cd* inside weirs is recognized as a complex hydraulic modeling challenge. The prediction of *Cd* is complicated due to the intricate interactions between the geometric parameters of the weir and the highly inconsistent hydraulic conditions and flow characteristics. Therefore, any prediction model employed needs to be robust to successfully handle these complexities and yield satisfactory predictions. Considering prior evaluations, the ELM-LWLR model has been found to effectively predict *Cd* values. This model leverages the strengths of both a global model (represented by ELM) and a local model (represented by LWLR-based radial basis kernel function). Using the LWLR approach in place of simple linear regression within the ELM model to create the ELM-LWLR model enhances the prediction accuracy. This improvement surpasses the accuracy of the classical ELM and LWLR models by up to 48.08% and exceeds the XGBoost model by 28.95%.

The ELM-LWLR model exhibits an outstanding superiority in prediction accuracy based on its *PBIAS*% value of −0.130%. The reported achievements indicate that the suggested model has a minor tendency to underestimate outcomes. The value obtained within − 0.130% is relatively close to zero, suggesting less bias compared to other models. When compared to MLR (*PBIAS* = −0.157%) and ELM (*PBIAS*% = −0.546%), ELM-LWLR performs better than ELM, which has a larger negative bias. Additionally, both XBGoost (*PBIAS* = 1.484%) and LWLR (*PBIAS* = 1.565%) exhibit significant positive biases which indicate a tendency to overestimate outcomes. Overall, ELM-LWLR stands out for its capability to balance complexity and accuracy, making it a reliable choice in scenarios where minimizing bias is crucial.

The suggested model (ELM-LWLR) exhibited excellent performance in predicting *Cd*, with an *R²* of 0.938. The results obtained showed that using the suggested hybrid model improves prediction accuracy. This aligns with previous findings where several researchers indicated that the prediction of *Cd* requires a strong model capacity. Furthermore, the predicted results presented in this paper are significantly higher than those obtained in the literature, which range from 0.16 to 0.92 (see Table 7). Accordingly, it can be said that the results of this study are largely consistent with those found in previous literature and shown in Table 7. The similarities can be summarized by stating that predicting *Cd* is a complex process and, therefore, requires a well-trained model to capture the significant information about the different flow patterns that affect *Cd*.

It can be observed from the reported results in the previous section that the classical model (ELM) generates a significantly larger amount of error compared to the developed model, even though the same structure, weights, and biases were used in this study. The results clearly indicate that the classical model’s reliance on a linear system for generating final outputs and predictions is its major weakness, leading to its inability to produce accurate predictions. The superiority of the ELM-LWLR model stems from its ability to leverage the strengths of both global and local modeling approaches. By incorporating the global model (ELM), which utilizes a nonlinear transfer function, alongside the localized, nonlinear technique of LWLR based on a radial basis kernel function, ELM-LWLR effectively enhances prediction accuracy. The proposed hybrid approach (ELM-LWLR) has a significant capability to adaptively adjust the output layer of ELM, resulting in improved performance when dealing with severe dynamic flow scenarios. The combination of these advanced approaches (ELM, and LWLR) ensures that ELM-LWLR captures complex patterns in the data, providing more precise results.

**Fig. 11:**
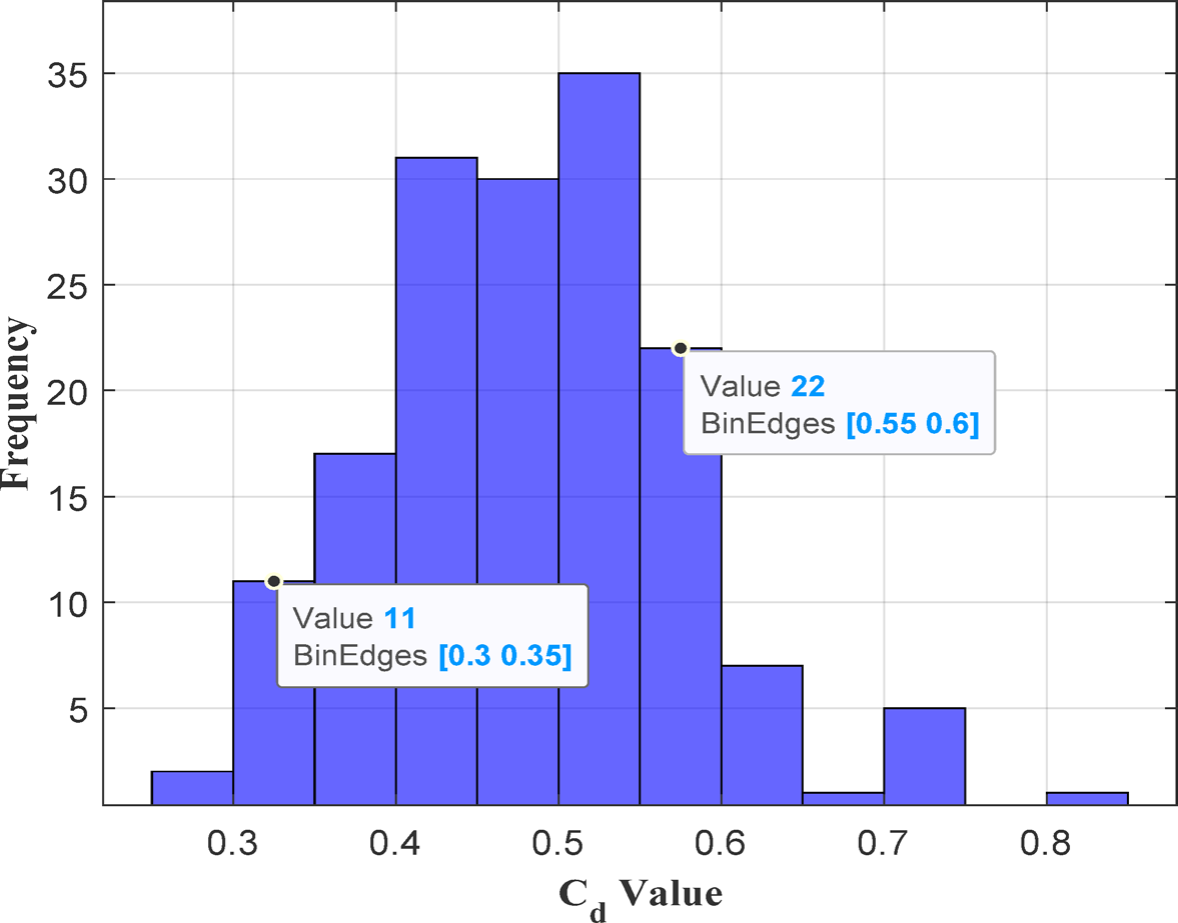
Histogram for explaining the distribution of Cd values.

One of the key advantages of the LWLR approach is it can handle non-linear relationships between variables, a common occurrence in many real-world datasets, as opposed to traditional linear regression which cannot handle such complexities. As demonstrated in Figs. 7 and 9, the ELM-LWLR model provides more accurate predictions for extreme *Cd* values compared to other models. The other comparable models do not provide excellent predictions for extreme *Cd* values, possibly due to insufficient training conditions where extreme *Cd* data is lacking. According to the Dot plot (see Fig. 6), the MLR model, for instance, is a simple model that confines its predictions within the range of 0.33 to 0.59. Similarly, most other models also produce predictions within this range. To visually analyze the data distribution and evaluate their frequencies, a histogram of *Cd* values is presented in Fig. 11. The *Cd* samples with frequencies exceeding 10 occurrences fall within the range of 0.30 to 0.35 for the first bin edge and 0.55 to 0.60 for the second bin edge. It is evident from this data that the frequency of extreme *Cd* values is low, leading most models, except the proposed model, to struggle to provide excellent predictions. Thus, the combination of ELM with LWLR in this proposed model generates highly accurate predictions for out-of-sample data points, thereby exhibiting excellent generalization capabilities for new data, especially in extreme values.

According to the results of the sensitivity analysis in this paper, two key features (*p*/*y*1 and *b*/*B*) influence the accuracy of the *Cd* prediction model. These features are crucial for capturing the underlying fluid dynamics, as their removal from the *Cd* calculation significantly reduces model performance. The *p*/*y*1 ratio is important because it directly affects the hydraulic head exerted on the weir, influencing flow rates and patterns. Furthermore, the ratio of the weir’s height to its length is critical for accurate *Cd* prediction; a higher weir height generally results in increased flow resistance and altered flow behavior. Additionally, the *b*/*B* ratio allows for a systematic evaluation of weir geometry, providing essential insights into how length differences affect discharge characteristics. Overall, the removal of these characteristics (e.g., *p*/*y*1 and *b*/*B*) leads to a significant loss in model accuracy, emphasizing their importance in capturing complex interactions in fluid dynamics.

The proposed ELM-LWLR model effectively addresses some of the issues found in previous methods for predicting *Cd* under varying flow conditions. For example, the traditional approaches which are shown in Table 7 often rely on empirical formulas or regression-based equations that depend on specific assumptions, leading to difficulties in generalizing across different gate types and flow scenarios. Accordingly, these adopted methods tend to provide lower prediction accuracy. However, the ELM-LWLR model leverages ELM and LWLR techniques, allowing it to capture complex, nonlinear relationships without rigid assumptions. Moreover, many prior ML models exhibit difficulties with generalization, particularly when trained on diverse datasets. Even advanced global models often perform poorly when applied to samples collected from various experimental sources. The ELM-LWLR model overcomes the mentioned limitation by combining the strengths of global modeling (ELM) and local modeling (through LWLR supported by RBF). The applied combination enables the ELM-LWLR model to effectively capture intricate local patterns, resulting in accurate predictions even with limited training data. Thus, the suggested approach excels at handling complicated hydraulic data by selecting high-performing outputs, exhibiting strong performance despite the limitations presented by small and diverse datasets.

The ELM-LWLR model provides substantial advantages for small industries that depend on precise hydraulic measurements for design and operational efficiency. By improving the accuracy of *Cd* predictions, the adopted model allows engineers to optimize side weir designs, thereby enhancing water management and resource allocation. Also, this enhancement is especially important for small-scale irrigation systems, wastewater management, and flood control, as accurate hydraulic calculations can significantly reduce costs by decreasing the reliance on physical tests and trial-and-error adjustments, improving performance, and fostering sustainability. Furthermore, the increased accuracy afforded by the ELM-LWLR model may diminish the necessity for physical tests and iterative adjustments, resulting in more effective water management and reliable flow control. Ultimately, the implementation of this model equips small industries to address complex engineering challenges with increased confidence and effectiveness, thereby driving better outcomes in their projects.

## Conclusion

The current research introduces a novel prediction model for estimating *Cd* by combining ELM with LWLR-based on RBF. The traditional ELM models depend on a linear regression system in their output layer, which can limit their effectiveness in addressing complex hydraulic engineering problems. Furthermore, classical ELM approaches function as global models, often struggling to adapt to local variations within the data. This can result in inefficiencies and decreased accuracy, particularly with datasets exhibiting diverse patterns. In this study, the LWLR has been utilized instead of the conventional linear system, thereby enhancing ELM’s ability to adjust to local data variations and improving its overall prediction performance. The suggested model (ELM-LWLR) is validated against traditional ELM, MLR, LWLR, and XGBoost. In the current paper, all the models were trained using different hydraulic and geometric parameters such as *F*1, *b*/*B*, b/y1, and *p*/*y*1.

The quantitative results demonstrate the observed superiority of the ELM-LWLR model, achieving higher accuracy prediction (*R*^*2*^ = 0.93, *d* = 0.984) and fewer estimation errors with *RMSE* of 0.027 and *MAE* of 0.018. Additionally, the distribution of the predicted *Cd* values obtained by ELM-LWLR is very similar to the experimental values, and the model generates a relatively low *MAPE* of 4.09% compared to other predictive models. The improvement in predictions achieved by the ELM-LWLR model is 37.21%, 28.95%, 48.08%, and 64.94% compared to LWLR, XGBoost, ELM, and MLR, respectively. Furthermore, this study explores the most effective parameters that have a substantial influence on *Cd* by conducting a sensitivity analysis. The analysis found that after removing the parameters *p*/*y*1 and *b*/*B*, the prediction accuracy is reduced significantly, indicating that these two parameters are crucial in the estimation of *Cd.*

The suggested ELM-LWLR model can be adapted for different hydraulic structures and flow conditions by customizing input features to include specific parameters, such as flow velocities and sediment transport. Furthermore, Optimizing the radial basis kernel or using alternative kernels can enhance accuracy, while adaptive learning techniques, like online learning, allow for responsiveness to changing conditions. Also, exploring ensemble methods and multiscale analysis may boost generalizability across diverse scenarios. Finally, advanced sensitivity analysis helps identify key factors, and synthetic datasets from computational fluid dynamics (CFD) can expand the training dataset. These enhancements enable effective analysis of complex hydraulic challenges in various applications.

This study recommends using bio-inspired optimization algorithms to tune the input weights of the proposed model, which may further enhance its accuracy and efficiency. Moreover, the suggested ELM-LWLR model can be adapted for various hydraulic structures and flow conditions by customizing its input features, such as incorporating specific parameters like flow velocities and sediment transport. Additionally, future research could focus on using the ELM-LWLR model to predict *Cd* for structures like V-notch and triangular labyrinth weirs. Also, testing the ELM-LWLR model with a wider range of datasets and different flow conditions, as well as investigation of synthetic datasets from computational fluid dynamics, could improve its robustness and adaptability. Thus, exploring extended data sets, optimizing the kernel function parameters would enhance its practical use in real-time dynamic systems and help address complex hydraulic challenges.

## Study limitations

The current paper is limited to a few experimental hydraulic datasets depending on about 160 samples. The limited data may hinder the efficiency of training the applied prediction models and thus reduce their generalization performance. Also, the study focuses only on rectangular sharp-crested side weirs and does not consider other weir types such as V-notch and orifice side, which have a different behavior under various hydraulic conditions. Consequently, the limitations regarding data size and type of the wire used in this research may limit the application of the proposed model.

The proposed ELM-LWLR model in this study demonstrated accurate predictions and offers several advantages, including a balanced approach between global and local modeling. It effectively handles diverse and complex flow conditions, delivering reliable predictions during the testing phase, even for scenarios not represented in the training data. However, it has some limitations, particularly in optimizing the *Tau* parameter, which significantly influences the performance of the ELM-LWLR model. The applied approach relies on a trial-and-error method for selecting the optimal *Tau*, which may impact model accuracy under different flow conditions. Future research should explore advanced optimization algorithms to fine-tune *Tau* and enhance predictive performance. Additionally, alternative kernel functions to the radial basis function could be investigated to improve model adaptability.

## Electronic supplementary material

Below is the link to the electronic supplementary material.

Supplementary Material 1

## Data Availability

The data are included in the paper and can be provided by the corresponding author upon request.

## Abbreviations

ANFIS: Adaptive Network-Based Fuzzy Inference System
ANN: Artificial neural network
*b*/*B*: Dimensionless weir length
b/y1: Ratio of weir length to the depth of upstream flow
*CA*: Combined Accuracy
Cd: Discharge coefficient
*d*: Willmott’s index of agreement
DE: Differential Evolution
ELM: Extreme Learning Machine
*ETR*: Extra Tree Regression
*F*1: Froude number
*FFNN*: Feedforward neural network
GA: Genetic Algorithm
GEP: Gene Expression Programming
GMDH: Group Method of Data Handling
GP: Gaussian Processes
GP: Genetic programming
GPR: Gaussian Process Regression
GRNN: Generalized regression neural networks
GWA: Grey Wolf algorithm
IWO: Invasive Weed Optimization
KELM: Kernel Extreme Learning Machine
KRR: Kernel Ridge Regression
LR: Linear Regression
LWLR: Locally Weighted Linear Regression
*MAE*: Mean absolute error
*MAPE*: Mean absolute percentage error
MLR: Multiple Linear Regression
*p*/*y*1: Ratio of weir height to length
*PBIAS*: percentage bias
PSO: Particle Swarm Optimization
R: Correlation coefficients
*R*^2^: Coefficient of determination
RF: Random forest
*RMSE*: Root mean square error
SA: Sensitivity analysis
SVM: Support vector regression
*U*_95_: Uncertainty analysis
XGBoost: Extreme Gradient Boosting
