# Supplementary material for: Integrated approach of extreme learning machines and locally weighted linear regression for improved discharge coefficient prediction
Source: Sci Rep. 2025 Jul 1;15:21761. doi: 10.1038/s41598-025-03812-z (PMC12216050; doi:10.1038/s41598-025-03812-z)
Supplement: Supplementary file 1 — Supplementary Material 1 [file 41598_2025_3812_MOESM1_ESM.docx]

| Model | Parameter | Data |
| --- | --- | --- |
| ELM | Hidden nodes | 20 |
|  | Transfer function | Log Sigmoid |
| ELM-LWR | Hidden nodes | 20 |
|  | Transfer function | Log Sigmoid |
|  | *Tau* | 0.1 |
| Xgboost | cost function | *RMSE* |
|  | Learning rate | 0.16 |
|  | Maximum depth of a tree | 7 |
|  | Subsample ratio of the training instance | 0.5 |
|  | Subsample ratio of columns when constructing each tree | 0.8 |
|  | gamma | 0.0005 |
|  | L2 | 1 |
|  | L1 | 0.1 |
|  | Minimum child weigh | 2 |

Appendix S: Tables

Table S1. Optimal hyperparameters utilized in this research.

Appendix K: Python codes

import pandas as pd
import numpy as np
import os

file_path = r'C:\Users\SD\Desktop\Mohammed Majeed\2024\Cd_coeffcients\data _Cd.xlsx'

# Read the Excel file into a DataFrame
df = pd.read_excel(file_path, sheet_name='Sheet1')

# Access the last column of the DataFrame
flowdata = df.iloc[:, :].values

flowdata = np.array(flowdata)

data = flowdata[:, 0:]
print(data[0])

p = 0.70

nD = round(p * len(data))

print(nD)
#
Tds = data # all data

# data shuffling
i12 = np.random.randint(1000)
np.random.seed(989)
print(i12)


rds = np.random.permutation(len(Tds))
print(np.max(rds))
Tds = Tds[rds,:]

print('*'*450)
print(Tds.shape[1])

Td = Tds[0:nD, :] # Training Data
# print(Td)


Ts = Tds[nD:] # Testing data
# print(Ts)
print(Td.shape[0])

# Data Normolazation

miv = np.min(Td, axis=0)
mav = np.max(Td, axis=0)

# print(mav)
# print(miv)

nTd = (Td - miv) / (mav - miv) # normolized train data
nTs = (Ts - miv) / (mav - miv) # normolized test data

xtrain = nTd[:, 0:-1]
ytrain = nTd[:, -1]

xtest = nTs[:, 0:-1]
ytest = nTs[:, -1]

################## XGboost ##################################
# Create DMatrix for XGBoost


ii1 = np.random.randint(10000)

dtrain = xgb.DMatrix(xtrain, label=ytrain)
dtest = xgb.DMatrix(xtest)
# dtest = xgb.DMatrix(X_test, label=Y_test)

# Set XGBoost parameters


params = {
 'objective': 'reg:squarederror', # Regression task
 'eval_metric': 'rmse', # Root Mean Squared Error
 'eta': 0.16, # Learning rate
 'max_depth': 7, # Maximum depth of a tree
 'subsample': 0.5, # Subsample ratio of the training instance
 'colsample_bytree': 0.8, # Subsample ratio of columns when constructing each tree
 'gamma': 0.0005, # Minimum loss reduction required to make a further partition on a leaf node
 'lambda': 1, # L2 regularization term on weights
 'alpha': 0.1, # L1 regularization term on weights
 'min_child_weight': 2, # Minimum sum of instance weight needed in a child
 'tree_method': 'auto', # Tree construction method (auto, exact, approx, hist, gpu_hist)
 'seed': 10 # Random seed for reproducibility
}

# Train the model
num_rounds = 1000 # Number of boosting iterations
model = xgb.train(params, dtrain, num_rounds)

# Make predictions on the test set
#
# predictions for Xgboost
y_pred1 = model.predict(dtrain)
y_pred2 = model.predict(dtest)

# ########################### MLR model #################################

# # Create and fit the MLR model
# model = LinearRegression()
# model.fit(xtrain, ytrain)
#
#
# y_pred1 =model.predict(xtrain)
# y_pred2 =model.predict(xtest)


# # # ############### ELM ########################
# #
# #
# def Mylogsig(x):
# x = x.astype(float)
# y = 1 / (1 + np.exp(-x))
# print(type(x))
# print(x)
#
#
# return y
#
# def myFA(x,w):
#
# a = np.ones((x.shape[0],1))
# bb = np.hstack((a,x))
# T = np.dot(bb,w)
# y = Mylogsig(T)
# return y
#
# #sigmoid Transfer Function
#
#
#
#
# # ii5 = np.random.randint(1000)
# # #
# # #
# # np.random.seed(830)
#
# # ELM Structure
# H = 20 # hidden nodes
# # Initialize the weights and bias
#
# w = 2 * np.random.rand(1 + xtrain.shape[1], H) - 1
#
# # creating hidden layer data
#
# HLt = myFA(xtrain, w) # training hidden layer
# HLs = myFA(xtest, w) # testing hidden layer
#
# o = np.linalg.pinv(HLt)
# ow = np.dot(o, ytrain.reshape(-1,1))
#
# # prediction
#
# y_pred1 = np.dot(HLt, ow) # for training
# y_pred2 = np.dot(HLs, ow) # for testing
# #
# #
# y_pred1 = np.squeeze(y_pred1)
# y_pred2 = np.squeeze(y_pred2)

# ################## LWLR #############################
#
# def LWLRF1(x, y, xqw, tau):
# ax = np.ones((x.shape[0], 1))
# X = np.hstack((ax, x))
# # print(X)
#
# aq = np.ones((xqw.shape[0], 1))
# XQ = np.hstack((aq, xqw))
# yf = []
# for i in range(XQ.shape[0]):
# xo = XQ[i]
#
# w1 = (X - xo) ** 2
#
# w3 = np.sum(w1, 1)
# w4 = np.exp(-w3 / (2 * tau * tau))
#
# B = np.linalg.pinv(X.T @ np.diag(w4) @ X) @ X.T @ np.diag(w4) @ y
# yf.append(xo @ B)
# return yf
#
#
#
#
#
# tau = 0.10
#
#
# y_pred1 = LWLRF1(xtrain, ytrain, xtrain, tau)
# y_pred2 = LWLRF1(xtrain, ytrain, xtest, tau)
#
#
#
# y_pred1 = np.squeeze(y_pred1)
# y_pred2 = np.squeeze(y_pred2)
